# Supplementary material for: Impact and cost-effectiveness of rotavirus vaccination in Niger: a modelling study evaluating alternative rotavirus vaccines
Source: BMJ Open. 2022 Oct 5;12(10):e061673. doi: 10.1136/bmjopen-2022-061673 (PMC9535195; doi:10.1136/bmjopen-2022-061673)

Table S1: ROSE trial cost of delivery data

**Cost categories**	**Unit cost**	**ROTARIX** (17.12 cm3)	**ROTAVAC** (4.2 cm3)	**ROTASIIL** (10.5 cm3)
International transportation and handling	$0.037 per cm3	$0.633	$0.155	$0.388
Vaccine distribution	$0.018 per cm3	$0.302	$0.074	$0.185
Cold chain storage	$0.010 per cm3	$0.167	$0.041	$0.102
Service delivery	$0.43 per dose	$0.43	$0.43	$0.43
**Total cost per dose**				
	**2018 US$**	**$1.53**	**$0.70**	**$1.11**
	**2021 US$**	**$1.65**	**$0.75**	**$1.20**

Table S2: ROSE trial cost of RVGE data (2021 US$)

Outpatient visits		Inpatient stay	
**Total sample**	**365**	**Total sample**	**26**
% girls	39.2%	% girls	34.6%
% boys	60.8%	% boys	65.4%
Average age	75.72 weeks	Average age	44.65 weeks
Average households' direct medical cost	$0.04	Average households' direct medical cost	$1.05
Average households' direct non-medical costs (food, transportation, day care expenses)	$1.03	Average households' direct non-medical costs (food, transportation, day care)	$3.59
Average indirect cost	$1.31	Average indirect cost	$5.65
**Average household costs**	**$2.37**	**Average household costs**	**$10.30**

Table S3: Parameters included in the probabilistic uncertainty analysis and their statistical distributions

(Base case values and range available from the main paper)

Parameter	Probability distribution	Source
**Population projections for the 2015 birth cohort**		
Population by single age/year between birth and 5·0 years	Beta-PERT (mid = UNPOP medium variant, range = UNPOP low/high variant)	United Nations Population Division. World Population Prospects - 2017 revision. Available from: https://population.un.org/wpp/ Accessed July 14, 2020.
**Disease burden estimates**		Isanaka S. et al. Rotavirus vaccine efficacy up to 2 years of age and against diverse circulating rotavirus strains in Niger: Extended follow-up of a randomized controlled trial. PLOS MEDICINE 2021 https://doi.org/10.1371/journal.pmed.1003655
RVGE incidence rate <5 years<br>Non-severe RVGE visits<br>Severe RVGE visits<br>Severe RVGE hospitalizations	Beta-PERT (mid and range reported in Table 1)	
Severe RVGE mortality rate <5 years	Beta-PERT (mid = Log transformed mean of 3 sources of estimates for Niger, range = 95% CI)	Clark A, Tate J, Parashar U, Jit M, Hasso-Agopsowicz M, Henschke N, et al. Mortality reduction benefits and intussusception risks of rotavirus vaccination in 135 low-income and middle-income countries: a modelling analysis of current and alternative schedules. Lancet Glob Heal. 2019 Nov 1;7(11):e1541–52. https://doi.org/10.1016/S2214-109X(19)30412-7
**Age distribution of RVGE deaths**		
Log Logistic scale parameter	Beta-PERT (mid = best fit for Niger/U5MR stratum, range = 95% CI for Niger/U5MR stratum)	Hasso-Agopsowicz M, Ladva CN, Lopman B, Sanderson C, Cohen AL, Tate JE, et al. Global Review of the Age Distribution of Rotavirus Disease in Children Aged under 5 Years Before the Introduction of Rotavirus Vaccination. Clin Infect Dis. 2019 Jan 28. https://doi.org/10.1093/cid/ciz060
**Disability weights for calcualting DALYs**		
Percentage of healthy time lost whilst living with the disease		
Non Severe RVGE	Beta-PERT (mid = Salomon, GBD 2013, moderate diarhea, range = Salomon, GBD 2013, moderate diarrhea, 95% uncetainty interval)	Salomon JA, Haagsma JA, Davis A, Maertens De Noordhout C, Polinder S, Havelaar AH, et al. Disability weights for the Global Burden of Disease 2013 study. Vol. 3, Articles Lancet Glob Health. 2015. https://doi.org/10.1016/S2214-109X(15)00069-8
Severe RVGE	Beta-PERT (mid = Salomon, GBD 2013, severe diarhea, range = Salomon, GBD 2013, severe diarhea, 95% uncetainty interval)	
Non Severe RVGE	Beta-PERT (mid = ROSE trial, range = ROSE trial)	Isanaka S. et al. Rotavirus vaccine efficacy up to 2 years of age and against diverse circulating rotavirus strains in Niger: Extended follow-up of a randomized controlled trial. PLOS MEDICINE 2021 https://doi.org/10.1371/journal.pmed.1003655
Severe RVGE	Beta-PERT (mid = ROSE trial, range = ROSE trial)	
**Vaccine coverage**		
Doses with DTP1, DTP2, DTP3	Beta-PERT (mid = WUENIC 2020, range = WUENIC 2020 +/-10%)	WHO UNICEF coverage estimates WHO World Health Organization: Immunization, Vaccines And Biologicals. Vaccine preventable diseases Vaccines monitoring system 2020 Global Summary Reference Time Series: DTP3. Available from https://apps.who.int/immunization_monitoring/globalsummary/timeseries/tswucoveragedtp3.html Accessed on accessed 15 September 2021.
**Vaccine timeliness**		
Log Logistic scale parameter	Beta-PERT (mid = best fit for Niger or schedule stratum, range = median age +/-10%)	Clark A, Sanderson C. Timing of children's vaccinations in 45 low-income and middle-income countries: an analysis of survey data. Lancet. 2009;373(9674):1543–9. https://doi.org/10.1016/s0140-6736(09)60317-2
**Vaccine efficacy**		
Initial efficacy against RVGE mortality (2wks after dose administered)	Beta (alpha = 723·19, beta = 98·31, [A] = 0%, [B] = 100%)	Clark A, van Zandvoort K, Flasche S, Sanderson C, Bines J, Tate J, Parashar U, Jit M. Efficacy of live oral rotavirus vaccines by duration of follow-up: a meta-regression of randomised controlled trials. The Lancet Infectious Diseases. 2019;19(7):717–727. https://doi.org/10.1016/S1473-3099(19)30126-4
Mean duration of vaccine efficacy in months	Beta-PERT	
Relative efficacy of 1 dose versus 2/3 doses	Base value: 0·63 (0·51%-0·79%)<br>Beta (alpha = 27·05, beta = 15·68, [A] = 0, [B] = 1)	

Parameter	Probability distribution	Source
**Vaccine program costs and healthcare costs**	Beta-PERT (mid=ROSE costing studies, range = ROSE costing studies)	ROSE costing studies<br>Gavi, the Vaccine Alliance. Co-financing Policy. Version 2.0. Available from https://www.gavi.org/news/document-library/gavi-co-financing-policy Accessed on 9 September 2021.<br>Gavi, The Vaccine Alliance. Eligibility and Transition Policy. Version 3.0. Available from https://www.gavi.org/news/document-library/gavi-eligibility-and-transition-policy Accessed on 9 September 2021.<br>Baral R. et al. Cost of illness for childhood diarrhea in low- and middle-income countries: a systematic review of evidence and modelled estimates. BMC Public Health. 2020; 20: 619. https://dx.doi.org/10.1186%2Fs12889-020-08595-8

Table S4: Health and economic benefits of vaccine (2021-2030, discounted), assuming 2-dose ROTARIX had similar impact to the 3-dose ROTAVAC or ROTASIIL.

**Health and economic benefits**	**ROTARIX (2 doses)**	**ROTAVAC (3 doses)**	**ROTASIIL (3 doses)**
Non-severe RVGE cases averted	774,058	774,058	774,058
Non-severe RVGE visits averted	394,770	394,770	394,770
Severe RVGE cases averted	556,379	556,379	556,379
Severe RVGE visits averted	283,753	283,753	283,753
Severe RVGE hospitalisations averted	140,263	140,263	140,263
Deaths averted	21,631	21,631	21,631
Healthcare cost averted (US$)			
Government perspective	5,062,897	5,062,897	5,062,897
Societal perspective	7,714,859	7,714,859	7,714,859
DALYs averted	544,142	544,142	544,142
Vaccine programme cost (US$)	77,257,820	46,670,448	61,805,765
**Cost-effectiveness ratio (US$ per DALYs averted compared to no vaccine scenario)**			
Government perspective	133	76	104
Societal perspective	128	72	99

Figure S1: Scenario analysis results showing incremental cost-effectiveness ratio (US$ per DALY averted) from the government and societal perspectives of ROTARIX, ROTAVAC, and ROTASIIL compared to no vaccination.

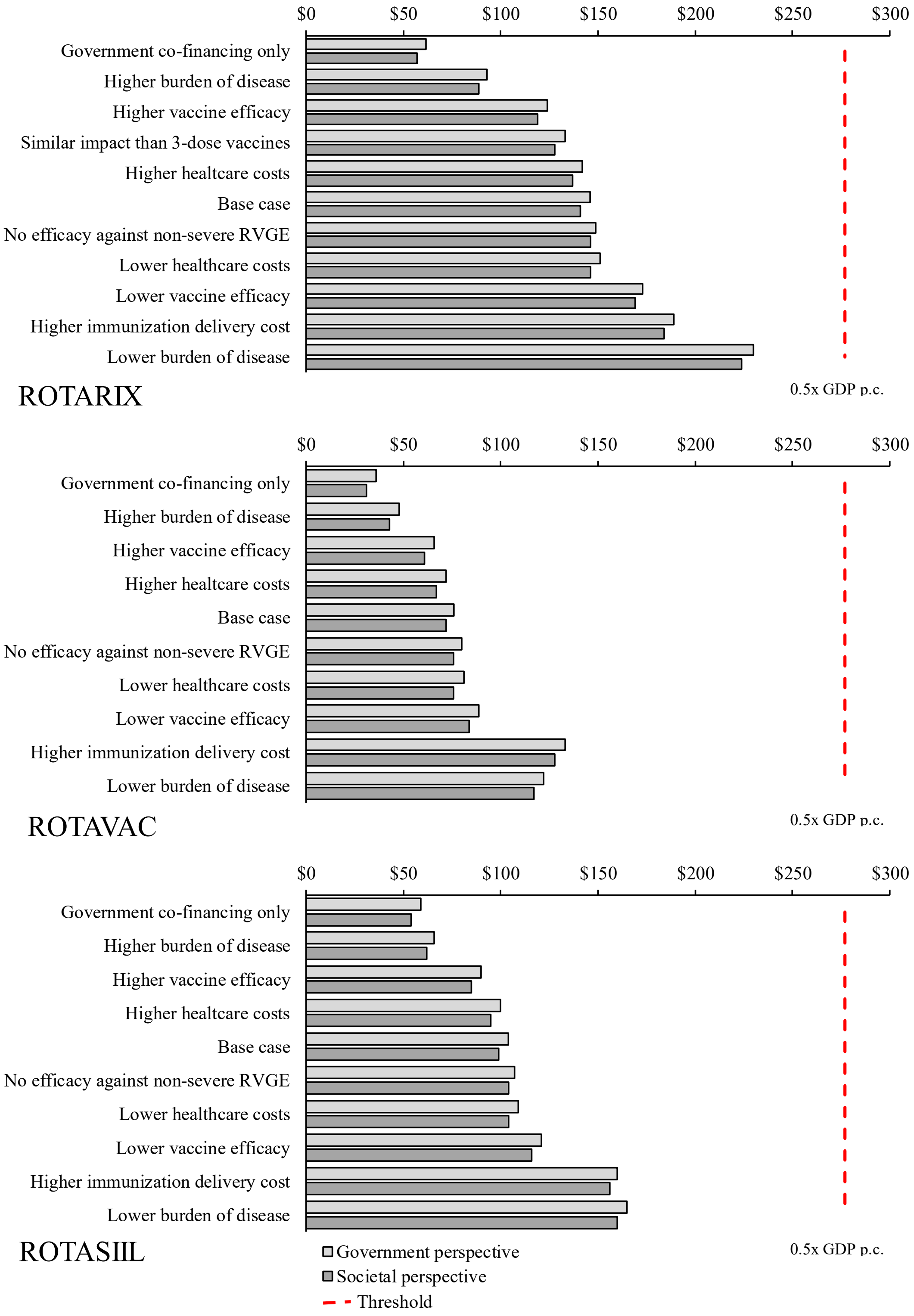

Supplement: Supplementary data [file bmjopen-2022-061673supp001.pdf]
