# Supplementary material for: Impact and cost-effectiveness of rotavirus vaccination in Niger: a modelling study evaluating alternative rotavirus vaccines
Source: BMJ Open. 2022 Oct 5;12(10):e061673. doi: 10.1136/bmjopen-2022-061673 (PMC9535195; doi:10.1136/bmjopen-2022-061673)
Supplement: Author's manuscript [file bmjopen-2022-061673.draft_revisions.pdf]

BMJ Open

BMJ Open is committed to open peer review. As part of this commitment we make the peer review history of every article we publish publicly available.

When an article is published we post the peer reviewers' comments and the authors' responses online. We also post the versions of the paper that were used during peer review. These are the versions that the peer review comments apply to.

The versions of the paper that follow are the versions that were submitted during the peer review process. They are not the versions of record or the final published versions. They should not be cited or distributed as the published version of this manuscript.

BMJ Open is an open access journal and the full, final, typeset and author-corrected version of record of the manuscript is available on our site with no access controls, subscription charges or pay-per-view fees (http://bmjopen.bmj.com).

If you have any questions on BMJ Open's open peer review process please email info.bmjopen@bmj.com

BMJ Open

Evaluating the potential impact and cost-effectiveness of alternative rotavirus vaccines in Niger

Journal:	*BMJ Open*
Manuscript ID	bmjopen-2022-061673
Article Type:	Original research
Date Submitted by the Author:	04-Feb-2022

Complete List of Authors:	Debellut, Frédéric ; PATH<br>Tang, Kevin; Epicentre; London School of Hygiene & Tropical Medicine<br>Clark, Andrew; London School of Hygiene and Tropical Medicine, Health Services Research and Policy<br>Pecenka, Clint; PATH<br>Assao, Bachir; Epicentre<br>Guindo, Ousmane; Epicentre<br>Grais, RF; Epicentre<br>Isanaka, Sheila; Epicentre; Harvard TH Chan School of Public Health, Departments of Nutrition and Global Health and Population

Keywords:	Health economics < HEALTH SERVICES ADMINISTRATION & MANAGEMENT, Health policy < HEALTH SERVICES ADMINISTRATION & MANAGEMENT, Public health < INFECTIOUS DISEASES, Paediatric infectious disease & immunisation < PAEDIATRICS, PREVENTIVE MEDICINE, PUBLIC HEALTH

SCHOLARONE™
Manuscripts

I, the Submitting Author has the right to grant and does grant on behalf of all authors of the Work (as defined in the below author licence), an exclusive licence and/or a non-exclusive licence for contributions from authors who are: i) UK Crown employees; ii) where BMJ has agreed a CC-BY licence shall apply, and/or iii) in accordance with the terms applicable for US Federal Government officers or employees acting as part of their official duties; on a worldwide, perpetual, irrevocable, royalty-free basis to BMJ Publishing Group Ltd ("BMJ") its licensees and where the relevant Journal is co-owned by BMJ to the co-owners of the Journal, to publish the Work in this journal and any other BMJ products and to exploit all rights, as set out in our licence.

The Submitting Author accepts and understands that any supply made under these terms is made by BMJ to the Submitting Author unless you are acting as an employee on behalf of your employer or a postgraduate student of an affiliated institution which is paying any applicable article publishing charge ("APC") for Open Access articles. Where the Submitting Author wishes to make the Work available on an Open Access basis (and intends to pay the relevant APC), the terms of reuse of such Open Access shall be governed by a Creative Commons licence – details of these licences and which Creative Commons licence will apply to this Work are set out in our licence referred to above.

Other than as permitted in any relevant BMJ Author's Self Archiving Policies, I confirm this Work has not been accepted for publication elsewhere, is not being considered for publication elsewhere and does not duplicate material already published. I confirm all authors consent to publication of this Work and authorise the granting of this licence.

For peer review only

Evaluating the potential impact and cost-effectiveness of alternative rotavirus vaccines in Niger

Authors

Frédéric Debellut[1], Kevin Tang[2,3], Andrew Clark[2], Clint Pecenka[4], Bachir Assao[5], Ousmane Guindo[5], Rebecca F. Grais[3], Sheila Isanaka[3,6]

1. Center for Vaccine Innovation and Access, PATH, Rue de Varembé 7, 1202 Geneva, Switzerland.

2. London School of Hygiene and Tropical Medicine, Keppel Street, London WC1E 7HT, United Kingdom.

3. Epicentre, Paris, France

4. Center for Vaccine Innovation and Access, PATH, 2201 Westlake Ave, Suite 200, Seattle, WA 98121, USA.

5. Epicentre, Niamey, Niger

6. Departments of Nutrition and Global Health and Population, Harvard T.H. Chan School of Public Health, 655 Huntington Avenue, Boston, MA, 02115 USA

Corresponding author: Frédéric Debellut fdebellut@path.org

For peer review only

Abstract

Objectives: To evaluate the cost-effectiveness of alternative rotavirus vaccines in Niger, using UNIVAC, a proportionate outcomes model.

Setting: The study leverages global, regional and local data to inform cost-effectiveness modelling. Local data were collected as part of a clinical trial taking place in the Madarounfa district, Maradi region, Niger.

Participants: The study models impact of infants vaccination on rotavirus gastroenteritis in children under five years of age.

Interventions: We compared the use of ROTARIX, ROTAVAC (Bharat Biotech, India), and ROTASIIL (Serum Institute, India) to no vaccination and to each other over a ten-year period starting in 2021.

Results: We estimated that ROTARIX, ROTAVAC, and ROTASIIL would each prevent 13 million cases and 20,000 deaths of children under five years over a ten-year period in Niger. Compared to no vaccination, the cost to avert a DALY was $146 with ROTARIX, $107 with ROTASIIL, and $76 with ROTAVAC from the government perspective. ROTAVAC dominated ROTARIX and ROTASIIL (e.g., provided similar or higher benefits at a lower cost) and had 90 percent chance to be cost-effective at a $100 willingness-to-pay threshold.

Conclusions: This study can inform decision-making around rotavirus vaccination policy in Niger, demonstrating that ROTAVAC is likely the most cost-effective option. Alternative products (ROTASIIL and ROTARIX) may also be considered by decision makers if they are priced more competitively, or if their cold chain requirements could bring additional economic benefits.

For peer review only

Strengths and limitations of this study

- This analysis uses a cost-effectiveness model, UNIVAC, developed to assist decision-makers in low- and middle-income countries in evaluating vaccine policy options.
- This is the first economic evaluation of rotavirus vaccination in Niger since its introduction in 2014, leveraging unique local data on cost to deliver vaccines, and cost borne by households for their children sick with rotavirus gastroenteritis.
- The study shows that evaluated vaccines, ROTARIX, ROTAVAC, and ROTASIIL, all avert one DALY for a cost below half of Niger's GDP per capita. ROTAVAC dominates other vaccines and has more than 90% probability of being cost-effective at a willingness-to-pay threshold of $100.
- Results provide decision-makers with evidence that may inform their future choice of rotavirus vaccine product. The availability of a variety of rotavirus vaccines since 2018 calls for a regular evaluation of all available options, including more recently prequalified vaccines.

For peer review only

Introduction

Rotavirus remains the leading cause of diarrhea mortality globally and was estimated to cause more than 150,000 deaths in children younger than five years of age in 2019, with more than 80% of these deaths occurring in sub-Saharan Africa.[1,2] The World Health Organization (WHO) recommends universal rotavirus vaccination for all children.[3]

Two live oral rotavirus vaccines (ROTARIX® [GlaxoSmithKline, Belgium] and RotaTeq® [Merck & Co., Inc., United States]) were prequalified and approved for global use by WHO in 2009.[4] One decade later, in 2018, two additional live oral rotavirus vaccines (ROTAVAC® [Bharat Biotech, India] and ROTASIIL® [Serum Institute, India]) entered the global market and received the same WHO prequalification status. These rotavirus vaccines are safe and effective and are used in more than 110 countries worldwide.[5,6]

In many of the world's poorest countries, including several African countries, rotavirus vaccines have been introduced with financial support from Gavi, the Vaccine Alliance. In 2014, the Republic of the Niger (hereafter referred to as Niger) introduced ROTARIX into its national immunization program with support from Gavi.[7] Prior to this, rotavirus was associated with around one-third of all severe diarrhea cases with moderate-to-severe dehydration in children aged under five years in the country.[8] The ROtavirus Safety and Efficacy (ROSE) clinical trial, implemented by Epicentre along with the Niger Ministry of Health and Médecins Sans Frontières, demonstrated the safety and efficacy of ROTASIIL in areas of Niger where ROTARIX was not yet available.[9,10] The ROSE clinical trial also collected data to estimate the cost of delivering rotavirus vaccine as part of the national immunization program as well as the costs borne by households when a child develops rotavirus illness.[10]

Despite inclusion of ROTARIX in the Expanded Programme on Immunization (EPI) program for several years and the availability of alternative rotavirus vaccines, there has been no formal economic evaluation of any rotavirus vaccine in Niger. This study aims to evaluate and compare the cost-effectiveness of

For peer review only

ROTARIX, ROTAVAC, and ROTASIIL using locally collected data. The results of this study can inform national decision-making on rotavirus vaccination policy in Niger.

Methods

Study design

We estimated the health impact and cost-effectiveness of three different rotavirus vaccines in Niger from 2021 to 2030 from the government and societal perspectives. We used an Excel proportionate outcomes model, UNIVAC (version 1.4.30), to compare three rotavirus vaccine products to no vaccination and to each other.[11] We evaluated the three rotavirus vaccines available through Gavi: ROTARIX, a monovalent human liquid vaccine available in a single-dose vial presentation and administered in a two-dose course; ROTAVAC, a monovalent human-bovine reassortant frozen liquid vaccine, available in a 5-dose vial presentation and administered in a three-dose course; and ROTASIIL, a pentavalent human-bovine reassortant lyophilized vaccine available in a 2-dose vial presentation and administered in a three-dose course.3 Gavi is an international organization aiming to increase equitable and sustainable access to vaccines for low- and middle-income countries. As a result, countries eligible for Gavi support can access negotiated prices from manufacturers through procurement with UNICEF. We did not evaluate RotaTeq as it is not available to Gavi-eligible countries and therefore would be unlikely considered for use in Niger by governmental authorities.[12]

The UNIVAC model was used to predict the number of averted rotavirus disease events in children under five years of age, related healthcare costs, and vaccination program costs. Input parameters were informed by data from the literature and primary data collected during the ROSE trial and its ancillary costing studies. The primary outcome measure is the incremental cost-effectiveness ratio (ICER) expressed as the cost in US$ per disability-adjusted life-year (DALY) averted. In the absence of a set willingness-to-pay (WTP) threshold in Niger, we interpret our cost-effectiveness results using 0.5 times the GDP per capita

For peer review only

(Niger's GDP per capita was US$553.9 in 2019[13]). All cost data are reported in 2021 USD, and we applied a standard 3% discount rate to all future costs and health outcomes.

Parent trial and ancillary costing studies

The ROSE trial was a double-blind, placebo-controlled randomized phase III trial that was implemented in the Madarounfa district in Niger from August 2014 to February 2018 (ClinicalTrials.gov identifier: NCT02145000). It assessed the efficacy and safety of ROTASIIL vaccine administered at 6, 10, and 14 weeks of age against rotavirus disease in healthy infants, demonstrating the safety of the vaccine and an efficacy level of 54.7% (95% CI 38.1% to 66.8%) at 24 months of follow-up.[10,14] Two ancillary costing studies were carried out alongside the trial to collect data on 1) the costs of immunization delivery in the national immunization program and 2) the costs of illness borne by households when they seek care for their child sick with rotavirus diarrhea.

For costs of immunization delivery, we included cost categories according to standard guidelines developed for the EPIC studies.[15] Primary data was collected from the central EPI at the national, regional, and district levels, as well as at four health facilities. Data collected covered international handling and transportation costs, national, regional, and district costs related to storing and distributing vaccines, the number of doses of vaccines stored and distributed, and the vaccines' presentation. This data was used to define a volume-based unit cost, which was then applied to each vaccine and their respective volume. We added costs at the health facility level, which was composed of labour costs to administer vaccines, storage, and transportation costs. Details on costs included for immunization delivery are available from supplementary Tables S1 and S2.

Healthcare costs of rotavirus gastroenteritis (RVGE) illness are estimated from primary data collected within the ROSE trial as well as published estimates. For the government perspective, we used estimates of direct medical costs of diarrhea as published by Baral *et al*.[16] For the societal perspective, direct medical costs were combined with household costs collected from a sub-group of caregivers presenting to

For peer review only

ROSE trial health facilities with a child sick with RVGE.[17] Household costs included out-of-pocket expenditures supported to seek care in terms of medicines, transportation, and food as well as indirect costs from time spent seeking care and caring for sick children. Caregiver time was valued using the GDP per capita divided by 365 and multiplied by the number of days spent seeking care or caring for a child in hospital. Data were collected from February 2018 to April 2019 from caregivers presenting to an outpatient health facility (n = 365) or whose child has been hospitalized (n = 26). Data was collected using standard questionnaires used to gather information on cost incurred by families during an episode.

All cost categories and line items included during this ancillary costing activities are available in the supplementary materials.

Rotavirus disease burden

We assumed that rotavirus infection would manifest as either non-severe RVGE or severe RVGE, aligning with classifications used in the trial. Non-severe RVGE resolves after seeking care in an outpatient setting or without seeking any care. Severe RVGE may resolve or be fatal, after seeking care in an outpatient or an inpatient setting or without seeking any care. Input parameters used for modeling rotavirus disease burden in Niger are available in Table 1. To define incidence rates for the different disease events, we used the incidence of severe and non-severe RVGE from the ROSE trial placebo population (6 weeks to 2 years of age) and converted this to incidence among children aged <5 years using data on the age distribution of rotavirus hospital admissions in high-mortality settings[18]. We defined incidence of RVGE clinic visits using the proportion of mothers reporting seeking care for their child sick with diarrhea (51%) based the most recently published DHS survey in Niger.[19] We used ROSE trial data to inform the incidence of severe RVGE hospitalizations, using the proportion of children with severe RVGE that were brought to the hospital (25%). We also used the ROSE trial data to inform the duration of severe and non-severe RVGE episodes. Lower and upper bounds for incidence reflect the low and high estimates used for Niger in previously published multi-country analyses.[20,21] The rate of RVGE deaths was taken from Clark *et al* (2019)[22] and represents the mean of three possible international estimates of

For peer review only

147 RVGE mortality in Niger. DALY weights were taken from the Global Burden of Disease (GBD) study,

148 using moderate and severe diarrhea as proxies for non-severe and severe RVGE.[23]

For peer review only

Table 1: Input parameters for estimation of disease burden

Input parameters	Base case	Lower bound	Upper bound	References
**Incidence (per 100,000 under-five children per year)**				
Non-severe RVGE cases	3,260	2,305	4,701	[10,18]
Non-severe RVGE visits	1,662	1,176	2,398	[19]
Severe RVGE cases	1,992	1,354	2,550	[10,18]
Severe RVGE visits	1,016	690	1,301	[19]
RVGE Hospitalisations	502	341	643	[10]
RVGE Deaths	115	74	176	[22]
**Duration of illness (days)**				
Non-severe RVGE case	2.36	2.13	2.59	[10]
Severe RVGE cases	3.58	3.07	4.09	[10]
**Disability weights**				
Non-severe RVGE case	0.188	0.125	0.264	[23]
Severe RVGE cases	0.247	0.164	0.348	
**Age distribution of disease events**				
**Age distribution**	**Cumulative percentage**			
< 1 month	0%			[18]
< 2 months	3%			
< 3 months	10%			
< 6 months	36%			
< 1 year	80%			
< 2 years	97%			
< 3 years	99%			
< 4 years	100%			
< 5 years	100%			

For peer review only

Vaccine efficacy, coverage, and coverage timeliness

Efficacy data for the three evaluated vaccines is taken from a meta-analysis pooling data from all published randomized controlled trials.[24] We assume that all products confer the same level of protection after the last dose of a full course, corresponding to 79% efficacy two weeks after the last dose, with subsequent waning bringing efficacy to 45% 12 months after the last dose.

We assume that vaccine efficacy against non-severe RVGE was 85% of the efficacy assumed for severe RVGE.[25] In an alternative scenario, we assumed 0% vaccine efficacy against non-severe disease, as can be derived from the ROSE trial, when comparing the reported efficacy against severe RVGE and efficacy against RVGE of any severity.[10]

Vaccine coverage and timeliness data for rotavirus vaccine doses 1, 2, and 3 were assumed to be the same as vaccine coverage and timeliness reported for diphtheria, tetanus, and pertussis (DTP) vaccine, where three doses of the vaccine are scheduled to be administered at 6, 10, and 14 weeks of age. For 2-dose ROTARIX, we only applied coverage for DTP1 and DTP2. Vaccine timeliness data were based on analyses by Clark *et al*.[26,22,27]

Vaccine prices and other procurement costs

We used vaccine prices published by Gavi, the Vaccine Alliance and assumed such prices would be available for the entire period of analysis.[28] In addition to benefitting from Gavi-negotiated prices, eligible countries only pay a share of vaccine procurement, dependent on their income per capita. In a scenario analysis, we applied the Niger co-financing share as an initial self-financing country per Gavi policy.[29,30] Other vaccine procurement related costs include safety boxes and vaccine wastage.

Vaccine wastage accounts for vaccine doses that are discarded, lost, damaged, or destroyed. Wastage is dependent on each vaccine presentation. We used wastage rates as recommended by Gavi's detailed product profiles and calculated with the WHO wastage rate tool. This resulted in a 4% wastage rate for

For peer review only

175 ROTARIX, 5% for ROTAVAC, and 9% for ROTASIIL.[28,31] All Input parameters used for estimation of
176 vaccine program costs and health care costs are available from Table 2.
177

For peer review only

Table 2: Input parameters for estimation of vaccine program costs and health care costs

Input parameter	Base case	Lower bound	Upper bound	Sources
**Vaccine coverage***				
Dose 1	92%	82.8%	100%	[26]
Dose 2	86.5%	77.9%	95.2%	
Dose 3 (ROTAVAC and ROTASIIL only)	81%	72.9%	89.1%	
**Vaccine price per dose (US$)**				
ROTARIX	$2.33	$0.20	-	[28]
ROTAVAC	$0.85	$0.13	-	
ROTASIIL	$0.95	$0.13	-	
**% Wastage**				
ROTARIX	4%	2%	6%	[28]
ROTAVAC	10%	5%	15%	
ROTASIIL	9%	5%	15%	
**Immunization delivery cost per dose (2021 US$)**				
ROTARIX	$1.65	$1	$2.77	ROSE costing study
ROTAVAC	$0.75	$0.75	$1.87	
ROTASIIL	$1.20	$1	$2.31	
**Health care cost (2021US$)**				
Unit cost of RVGE visit<br>Government perspective	$4.79	$2.40	$7.19	[16] and ROSE costing study for households' costs
Societal perspective	$7.16	$4.77	$9.56	
Unit cost of severe RVGE hospitalisation<br>Government perspective	$18.38	$9.19	$27.57	
Societal perspective	$28.68	$19.49	$37.87	

* For vaccine timeliness we assume that by 6 months of age, 91% of children would have received their first dose, 80% would have received their second dose, and, when applicable, 60% would have received their third dose of rotavirus vaccine. Data were fitted using a gamma curve to estimate timeliness by week of age <5 years.

For peer review only

Sensitivity analyses

To account for uncertainty, we undertook deterministic and probabilistic uncertainty analyses. Deterministic analyses covered a series of scenarios more and less favorable to the vaccine. In scenario 1, we accounted only for the country co-financing Niger currently pays for vaccine procurement; in scenario 2, we accounted for a higher burden of disease; in scenario 3, we accounted for a lower burden of disease; in scenario 4, we accounted for a higher vaccine efficacy; in scenario 5, we assumed a lower vaccine efficacy; in scenario 6, we accounted for higher healthcare costs; in scenario 7, for lower healthcare costs; in scenario 8, for higher immunization delivery costs; and in scenario 9, we assumed vaccines had no efficacy against non-severe RVGE. Finally, as our assumptions around similar efficacy of the last dose of vaccine led to lower impact of the 2-dose ROTARIX vaccine compared to 3-dose ROTAVAC and ROTASIIL vaccines, we also evaluated the cost-effectiveness of ROTARIX with similar impact to the 3-dose vaccines (scenario 10).

We ran a probabilistic uncertainty analysis for the three vaccines. We conducted 1,000 model runs sampling input data and statistical distributions available in supplementary table S3. We reported, for each vaccine, the mean ICER value from the government perspective as well as results for each model run. We also built a cost-effectiveness acceptability curve showing the probability for the dominant vaccine to be cost-effective at different WTP thresholds.

No Patient and Public Involvement

This research was done without patient or public involvement. It was not appropriate or possible to involve patients or the public in the design, or conduct, or reporting, or dissemination of our research as the study focused on assessing cost and cost-effectiveness of an intervention provided by the Ministry of Health.

For peer review only

Results

Deterministic analysis

We estimate that a vaccination program using any of the three rotavirus vaccines analysed could avert around 13 million RVGE cases and 20,000 deaths over 10 years in Niger. With 660,000 visits and 136,000 hospitalizations averted, rotavirus vaccination could result in healthcare cost savings of US$5 million from the government and US$7 million from the societal perspective. Vaccination program costs vary depending on which vaccine is used: US$46.7 million with ROTAVAC, US$61.8 million with ROTASIIL, and US$77.3 with ROTARIX, when not accounting for Gavi subsidies for vaccine procurement. Detailed figures on outcomes related to each vaccine are available in Table 3. The cost per DALY averted also varies by vaccine and perspective. From the government perspective, and when comparing each vaccine to 'no vaccine', the most favourable ICER is with ROTAVAC (US$76 per DALY averted), followed by ROTASIIL (US$104) and then ROTARIX (US$146). From the societal perspective, the same order remains, with ROTAVAC having the lowest ICER (US$72), followed by ROTASIIL (US$99) and ROTARIX (US$141). When assuming 2-dose ROTARIX had similar impact to the 3-dose ROTAVAC or ROTASIIL, the ROTARIX ICER decreased to $133 from the government perspective and $128 from the societal perspective (Table S4).

ROTAVAC can be considered cost-effective based on a WTP threshold set at half the GDP per capita (US$277). Both ROTARIX and ROTASIIL may not be cost-effective given the availability of ROTAVAC; i.e., if ROTARIX is compared directly to ROTAVAC, the cost per DALY averted would be US$638, and if ROTASIIL is compared directly to ROTAVAC, health benefits are the same, but vaccination program costs are higher (ICER is undefined). However, there is considerable uncertainty around several of our inputs and the rank order would change if there were changes in important variables.

For peer review only

232 **Table 3: Health and economic benefits of vaccine (2021-2030, discounted)**

**Health and economic benefits**	**ROTARIX (2 doses)**	**ROTAVAC (3 doses)**	**ROTASIIL (3 doses)**
Non-severe RVGE cases averted	706,843	774,058	774,058
Non-severe RVGE visits averted	360,490	394,770	394,770
Severe RVGE cases averted	508,066	556,379	556,379
Severe RVGE visits averted	259,113	283,753	283,753
Severe RVGE hospitalisations averted	128,083	140,263	140,263
Deaths averted	19,752	21,631	21,631
Healthcare cost averted (US$)			
Government perspective	4,623,393	5,062,897	5,062,897
Societal perspective	7,045,141	7,714,859	7,714,859
DALYs averted	496,905	544,142	544,142
Vaccine programme cost (US$)	77,257,820	46,670,448	61,805,765
**Cost-effectiveness ratio (US$ per DALYs averted compared to no vaccine scenario)**			
Government perspective	146	76	104
Societal perspective	141	72	99

233

234 *Scenario analysis*

235 The scenario analysis shows that all three rotavirus vaccines have cost-effectiveness ratios below the half
236 GDP per capita threshold (Figure S1). The most favourable scenario is the scenario where we account for
237 the level of co-financing currently supported by the country. When accounting for vaccine co-financing
238 instead of the Gavi price from the government perspective, ROTAVAC has a cost-effectiveness ratio of
239 US$36, ROTASIIL US$59, and ROTARIX US$62. The least favourable scenario is the scenario where
240 we account for a lower burden of disease. In this scenario, from the government perspective, the cost to
241 avert a DALY is US$122 with ROTAVAC, US$165 with ROTASIIL, and US$230 with ROTARIX.

242 *Probabilistic sensitivity analysis*

For peer review only

243 Cost-effectiveness of rotavirus vaccinination in Niger is further confirmed by the probabilistic uncertainty
244 analysis whereby all 1,000 runs returned cost-effectiveness ratio values below the assumed threshold. The
245 same order of the vaccines' cost-effectiveness ratios remains, with ROTAVAC dominating other options
246 (less costly and better health outcome) at a mean ICER of US$73 per DALY averted, ROTASIIL at
247 US$103, and ROTARIX at US$132 (Figure 1). The cost-effectiveness acceptability curve shows the high
248 probablity for the dominant vaccine option to be cost-effective at thresholds lower than $277. ROTAVAC
249 has >99% chance of being cost-effective at a WTP threshold of $150 (Figure 2).

250

251

For peer review only

Discussion

This analysis confirms that rotavirus vaccine averts a substantial burden in Niger and remains cost-effective several years after introduction. All vaccines evaluated have ICERs below half of the GDP per capita WTP threshold, despite each product's differences in presentation and characteristics. Cost-effectiveness ratios for the vaccines are generally of similar scale, with ROTAVAC dominating other options. ROTAVAC's economic advantage is mainly driven by its lower volume resulting in lower immunization delivery cost. This finding is consistent across all scenario and probabilistic analyses and remains true even in the current context with Niger paying a share of the vaccine price through Gavi co-procurement. This study demonstrates that product presentations and their associated costs should be evaluated closely.

There are limits to this analysis worth highlighting. The immunization cost of delivery values used in the study are calculated based on the volume of each product. This advantages the product with the lowest cold chain footprint. Other factors should be considered when evaluating or revaluating product selection. In our example, policymakers may wish to consider the feasibility of using a product with a smaller cold chain footprint that also must be stored in the negative cold chain at central level. ROTASIIL, though a higher volume product, could be, under certain circumstances, stored outside of the cold chain. If use of such a thermostable product was feasible country-wide in Niger without incurring additional costs, then ROTASIIL would potentially become the best choice from an economic perspective. Additional costs may be incurred from having to develop separate processes for an individual vaccine. Experiences to date with use of vaccines outside of the cold chain, through the Controlled Temperature Chain (CTC) protocol, have led to positive outcomes in campaign settings. This approach is, however, not currently recommended for routine vaccines.[32]

The ROSE trial provided unique primary data on cost of delivery and households' cost of illness. Immunization cost of delivery data are coming from a limited number of facilities, from one region only. While we address this limitation through sensitivity analysis, the immunization delivery cost data would

For peer review only

benefit from being based on a higher set of facilities and additional regions and districts. The same limitation applies to the cost of illness data used in the modelling. The small sample for hospitalized children was collected from a single facility within the ROSE trial.

Different impact values with 2-dose versus 3-dose vaccines are based on how the model calculates health outcomes based on efficacy values and waning of protection after the last dose of vaccine. The third dose of ROTAVAC and ROTASIIL is given 4 weeks later and thus translates in a small delay in the waning of protection compared with ROTARIX. This in turns results in a slightly higher number of averted cases, visits, hospitalizations, and deaths. This has not been proven empirically, and studies that have assessed the impact of additional doses of vaccines have reported inconsistent results.[33] However, this is unlikely to impact our findings as, when accounting for similar impact among the three vaccines, the ROTARIX ICER remains inferior of that of ROTAVAC or ROTASIIL ($133 per DALY averted from the government perspective).

This evaluation does not account for indirect effects of vaccination. However, considering recent work by *Ferrari et al*., it looks like indirect effects may not be a key driver in Niger and that our static model may provide reasonable results.[34]

Rotarix was included in the national immunization program in Niger in 2014, but no economic evaluation was conducted at the time. In 2014 only two vaccine presentations were available (ROTARIX and RotaTeq). At end of 2021, there are more than 10 rotavirus vaccine presentations that countries can choose from, making continued analysis essential to make informed choices.

The findings of this study can inform decision-making around rotavirus vaccination policy in Niger, demonstrating that ROTAVAC is likely to be the most cost-effective option. Alternative products (ROTASIIL and ROTARIX) may also be considered by decision makers if they can be priced more competitively, or if their cold chain requirements could bring additional economic benefits. Regular re-

For peer review only

evaluation is likely to be required as more evidence emerges on the relative costs and benefits of each product.

Figure 1: Mean ICER and probabilistic cost-effectiveness results from the probabilistic uncertainty analysis, government perspective, over a 10-year period

Figure 2: Cost-effectiveness acceptability curve for the dominant option (ROTAVAC)

For peer review only

Data availability statement

The authors confirm that the data supporting the findings of this study are available within the article and its supplementary materials. The model used is available online at https://www.paho.org/en/provac-toolkit

ROSE trial data: The deidentified dataset supporting this research can be made available following a submitted request as per Epicentre and General Data Protection Regulation (EU) 2016/679 data sharing policy. Additional information is available at https://epicentre.msf.org/en/data-access-request.

Funding statement

This work was supported, in whole or in part, by the Bill & Melinda Gates Foundation [OPP1147721]. Under the grant conditions of the Foundation, a Creative Commons Attribution 4.0 Generic License has already been assigned to the Author Accepted Manuscript version that might arise from this submission.

ROSE trial funding: Funding was provided to Epicentre by Médecins Sans Frontières (MSF)-Operational Center Geneva (https://www.msf.org/) and the Kavli Foundation, Norway (https://www.kavlifoundation.org/). Serum Institute of India Pvt Limited provided vaccine and placebo in kind.

Ethics approval statement

Ethical assessment was undertaken, and approval given, by the Comité National d'Ethique pour la Recherche en Santé, CNERS, Ministère de la Santé Publique, République du Niger. Délibération N°28/2018/CNERS.

Contributorship statement

FD, KT, CP and SI designed the study. FD, KT, BA and OG collected and analyzed the data. AC developed the study model. CP, AC, RG, SI provided technical inputs to the study. FD ran the analysis and wrote the first draft of the manuscript. All authors reviewed and approved the final manuscript.

For peer review only

331 **Conflict of interest statement**

332 The authors have no conflict to report.

For peer review only

References

[1] Troeger C, Forouzanfar M, Rao PC, Khalil I, Brown A, Reiner RC, Fullman N, Thompson RL, Abajobir A, Ahmed M, et al. Estimates of global, regional, and national morbidity, mortality, and aetiologies of diarrhoeal diseases: a systematic analysis for the Global Burden of Disease Study 2015. The Lancet Infectious Diseases. 2017;17(9):909–948. doi:10.1016/S1473-3099(17)30276-1

[2] GBD Results Tool. Institute for Health Metrics and Evaluation. Available from http://ghdx.healthdata.org/gbd-results-tool Accessed on 7 April 2021.

[3] WHO. Rotavirus vaccines: WHO position paper – July 2021. Weekly Epidemiological Record, N°28, 16 July 2021. Available from https://apps.who.int/iris/handle/10665/346571 Accessed on 20 October 2019.

[4] WHO. Summary of Key Characteristics of WHO Prequalified Rotavirus Vaccines. WHO/IVB/2021.03. Available from file:///C:/Users/fdebellut/Downloads/WHO-IVB-2021.03-eng%20(1).pdf Accessed on 21 October 2021.

[5] Bergman H, Henschke N, Hungerford D, Pitan F, Ndwandwe D, Cunliffe N, Soares-Weiser K. Vaccines for preventing rotavirus diarrhoea: vaccines in use. Cochrane Database of Systematic Reviews 2021, Issue 11. Art. No.: CD008521. https://doi.org/10.1002/14651858.CD008521.pub6

[6] ViewHub. International Vaccine Access Center (IVAC). Available from https://view-hub.org/ Accessed on 25 March 2021.

[7] JSI Research and Training Institute Inc. Lessons Learned: Rotavirus & PCV Vaccine Introduction in Niger. March 2016. Available from https://publications.jsi.com/JSIInternet/Inc/Common/_download_pub.cfm?id=17995&lid=3 Accessed on 21 October 2021.

[8] Page A., Jusot V., Mamaty A., et al. Rotavirus Surveillance in Urban and Rural Areas of Niger, April 2010–March 2012. Emerging Infectious Diseases. 2014 Apr; 20(4): 573–580. https://dx.doi.org/10.3201%2Feid2004.131328

[9] Isanaka S., Guindo O., Langendorf C., et al. Efficacy of a Low-Cost, Heat-Stable Oral Rotavirus Vaccine in Niger. New England Journal of Medicine 2017; 376:1121-1130

[10] Isanaka S. et al. Rotavirus vaccine efficacy up to 2 years of age and against diverse circulating rotavirus strains in Niger: Extended follow-up of a randomized controlled trial. PLOS MEDICINE 2021 https://doi.org/10.1371/journal.pmed.1003655

[11] PAHO. ProVac Toolkit. Tools. About UNIVAC. Available from https://www.paho.org/en/provac-toolkit Accessed on 03 December 2021.

[12] NPR. Merck Pulls Out Of Agreement To Supply Life-Saving Vaccine To Millions Of Kids. November 1, 2018. Available from https://www.npr.org/sections/goatsandsoda/2018/11/01/655844287/merck-pulls-out-of-agreement-to-supply-life-saving-vaccine-to-millions-of-kids?t=1638525408118 Accessed on 03 December 2021.

[13] World Bank national accounts data, and OECD National Accounts data files. GDP per capita (current US$) – Niger. Available from https://data.worldbank.org/indicator/NY.GDP.PCAP.CD?locations=NE Accessed on 1 September 2021.

[14] Coldiron M., Guindo O., Makarimi R., et al. Safety of a heat-stable rotavirus vaccine among children in Niger: Data from a phase 3, randomized, double-blind, placebo-controlled trial. Vaccine. Volume 36, Issue 25, 14 June 2018, Pages 3674-3680. https://doi.org/10.1016/j.vaccine.2018.05.023

For peer review only

[15] Brenzel L., Young D., Walker D.G. Costs and financing of routine immunization: Approach and selected findings of a multi-country study (EPIC). Vaccine.2015 May 7;33 Suppl 1:A13-20. https://doi.org/10.1016/j.vaccine.2014.12.066

[16] Baral R. et al. Cost of illness for childhood diarrhea in low- and middle-income countries: a systematic review of evidence and modelled estimates. BMC Public Health. 2020; 20: 619. https://dx.doi.org/10.1186%2Fs12889-020-08595-8

[17] World Health Organization. (2005). Guidelines for estimating the economic burden of diarrhoeal disease, with focus on assessing the costs of rotavirus diarrhoea. World Health Organization. https://apps.who.int/iris/handle/10665/69137

[18] Hasso-Agopsowicz H. Global Review of the Age Distribution of Rotavirus Disease in Children Aged <5 Years Before the Introduction of Rotavirus Vaccination. *Clinical Infectious Diseases*, Volume 69, Issue 6, 15 September 2019, Pages 1071–1078, https://doi.org/10.1093/cid/ciz060

[19] Institut National de la Statistique (INS) et ICF International, 2013. Enquête Démographique et de Santé et à Indicateurs Multiples du Niger 2012. Calverton, Maryland, USA : INS et ICF International. Available from https://dhsprogram.com/pubs/pdf/FR277/FR277.pdf Accessed 1 September 2021.

[20] Debellut F. et al. Re-evaluating the potential impact and cost-effectiveness of rotavirus vaccination in 73 Gavi countries: a modelling study. The Lancet Global Health 2019. https://doi.org/10.1016/S2214-109X(19)30439-5

[21] Debellut F. et al. Evaluating the potential economic and health impact of rotavirus vaccination in 63 middle-income countries not eligible for Gavi funding: a modelling study. Lancet Global Health, 2021, https://doi.org/10.1016/S2214-109X(21)00167-4

[22] Clark A. et al. Mortality reduction benefits and intussusception risks of rotavirus vaccination in 135 low-income and middle-income countries: a modelling analysis of current and alternative schedules. Lancet Global Health, 2019, https://doi.org/10.1016/S2214-109X(19)30412-7

[23] Salomon J. A. Disability weights for the Global Burden of Disease 2013 study. Lancet Glob Health. 2015 Nov;3(11):e712-23. https://doi.org/10.1016/s2214-109x(15)00069-8

[24] Clark A, van Zandvoort K, Flasche S, Sanderson C, Bines J, Tate J, Parashar U, Jit M. Efficacy of live oral rotavirus vaccines by duration of follow-up: a meta-regression of randomised controlled trials. The Lancet Infectious Diseases. 2019;19(7):717–727. https://doi.org/10.1016/S1473-3099(19)30126-4

[25] Rogawski ET, Platts-Mills JA, Ross Colgate E, Haque R, Zaman K, Petri WA, et al. Quantifying the Impact of Natural Immunity on Rotavirus Vaccine Efficacy Estimates: A Clinical Trial in Dhaka, Bangladesh (PROVIDE) and a Simulation Study. J Infect Dis Nat Immun Vaccine Effic. JID. 2018:861. https://doi.org/10.1093/infdis/jix668

[26] WHO UNICEF coverage estimates WHO World Health Organization: Immunization, Vaccines And Biologicals. Vaccine preventable diseases Vaccines monitoring system 2020 Global Summary Reference Time Series: DTP3. Available from https://apps.who.int/immunization_monitoring/globalsummary/timeseries/tswucoveragedtp3.html Accessed on accessed 15 September 2021.

[27] Clark A, Sanderson C. Timing of children's vaccinations in 45 low-income and middle-income countries: an analysis of survey data. Lancet. 2009;373(9674):1543–9. https://doi.org/10.1016/s0140-6736(09)60317-2

[28] Gavi, The Vaccine Alliance. Product information for vaccines and cold chain equipment. Detailed product profiles. Available from https://www.gavi.org/news/document-library/detailed-product-profiles Accessed on 9 September 2021

For peer review only

[29] Gavi, the Vaccine Alliance. Co-financing Policy. Version 2.0. Available from https://www.gavi.org/news/document-library/gavi-co-financing-policy Accessed on 9 September 2021.

[30] Gavi, The Vaccine Alliance. Eligibility and Transition Policy. Version 3.0. Available from https://www.gavi.org/news/document-library/gavi-eligibility-and-transition-policy Accessed on 9 September 2021.

[31] WHO. Vaccine Wastage Rates Calculator. 2021. Available from https://www.who.int/publications/m/item/vaccine-wastage-rates-calculator Accessed on 19 October 2021.

[32] World Health Organization. Health Products and Policy Standards. Controlled Temperature Chain (CTC). Beyond the traditional cold chain. Available from https://www.who.int/teams/health-product-and-policy-standards/standards-and-specifications/ctc/ Accessed on 29 October 2021.

[33] Lee B. Update on rotavirus vaccine underperformance in low- to middle-income countries and next-generation vaccines. Human Vaccines Immunotherapeutics 2021; 17(6): 1787–1802. https://dx.doi.org/10.1080%2F21645515.2020.1844525

[34] Park J., Goldstein J., Haran M., and Ferrari M. An ensemble approach to predicting the impact of vaccination on rotavirus disease in Niger. Vaccine. 2017 Oct 13; 35(43): 5835–5841. https://dx.doi.org/10.1016%2Fj.vaccine.2017.09.020

For peer review only

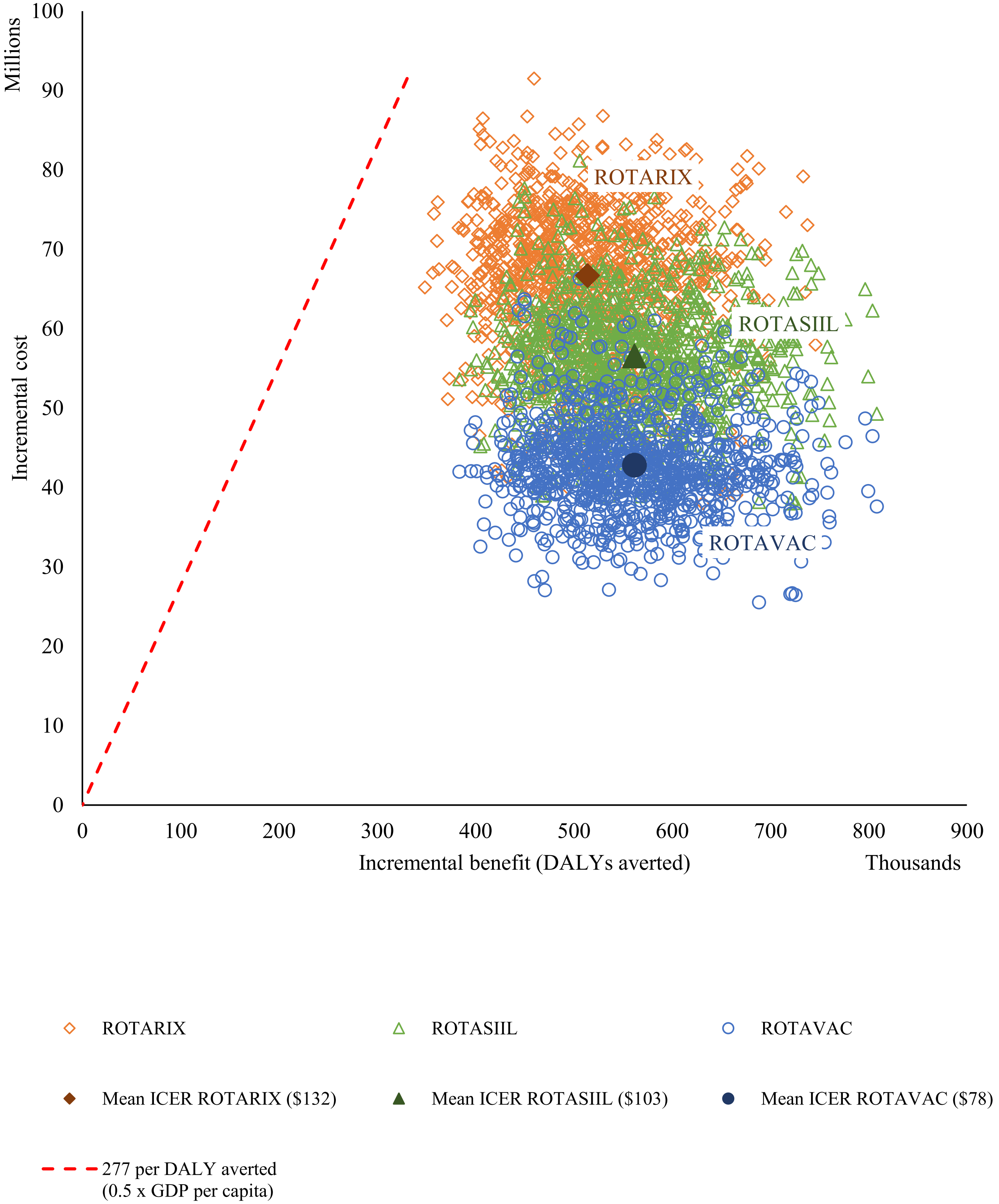
Millions
Incremental cost
100
90
80
70
60
50
40
30
20
10
0
0
100
200
300
400
500
600
700
800
900
Incremental benefit (DALYs averted)
Thousands
ROTARIX
ROTASIIL
ROTAVAC
ROTARIX
ROTASIIL
ROTAVAC
Mean ICER ROTARIX ($132)
Mean ICER ROTASIIL ($103)
Mean ICER ROTAVAC ($78)
277 per DALY averted
(0.5 x GDP per capita)

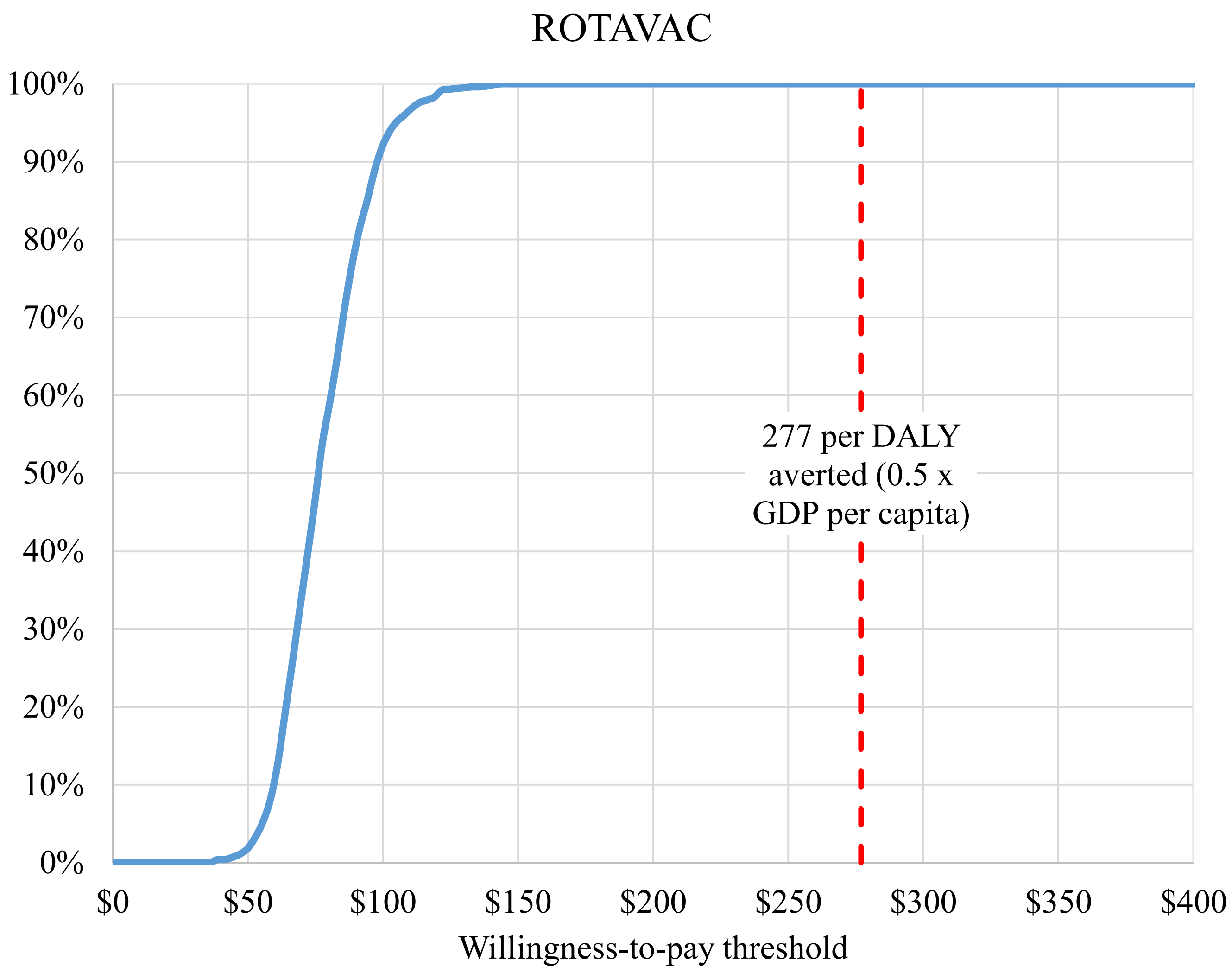
ROTAVAC
100%
90%
80%
70%
60%
50%
40%
30%
20%
10%
0%
$0
$50
$100
$150
$200
$250
$300
$350
$400
Willingness-to-pay threshold
277 per DALY averted (0.5 x GDP per capita)

Table S1: ROSE trial cost of RVGE data (2021 US$)

Outpatient visits		Inpatient stay	
**Total sample**	**365**	**Total sample**	**26**
% girls	39.2%	% girls	34.6%
% boys	60.8%	% boys	65.4%
Average age	75.72 weeks	Average age	44.65 weeks
Average households' direct medical cost	$0.04	Average households' direct medical cost	$1.05
Average households' direct non-medical costs (food, transportation, day care expenses)	$1.03	Average households' direct non-medical costs (food, transportation, day care)	$3.59
Average indirect cost	$1.31	Average indirect cost	$5.65
**Average household costs**	**$2.37**	**Average household costs**	**$10.30**

For peer review only

Table S2: ROSE trial cost of delivery data

Cost categories	Unit cost	ROTARIX (17.12 cm3)	ROTAVAC (4.2 cm3)	ROTASIIL (10.5 cm3)
International transportation and handling	$0.037 per cm3	$0.633	$0.155	$0.388
Vaccine distribution	$0.018 per cm3	$0.302	$0.074	$0.185
Cold chain storage	$0.010 per cm3	$0.167	$0.041	$0.102
Service delivery	$0.43 per dose	$0.43	$0.43	$0.43
**Total cost per dose**				
	**2018 US$**	**$1.53**	**$0.70**	**$1.11**
	**2021 US$**	**$1.65**	**$0.75**	**$1.20**

For peer review only

Table S3: Parameters included in the probabilistic uncertainty analysis and their statistical distributions

(Base case values and range available from the main paper)

Parameter	Probability distribution	Source
**Population projections for the 2015 birth cohort**		
Population by single age/year between birth and 5·0 years	Beta-PERT (mid = UNPOP medium variant, range = UNPOP low/high variant)	United Nations Population Division. World Population Prospects - 2017 revision. Available from: https://population.un.org/wpp/ Accessed July 14, 2020.
**Disease burden estimates**		Isanaka S. et al. Rotavirus vaccine efficacy up to 2 years of age and against diverse circulating rotavirus strains in Niger: Extended follow-up of a randomized controlled trial. PLOS MEDICINE 2021 https://doi.org/10.1371/journal.pmed.1003655
RVGE incidence rate <5 years<br>Non-severe RVGE visits<br>Severe RVGE visits<br>Severe RVGE hospitalizations	Beta-PERT (mid and range reported in Table 1)	
Severe RVGE mortality rate <5 years	Beta-PERT (mid = Log transformed mean of 3 sources of estimates for Niger, range = 95% CI)	Clark A, Tate J, Parashar U, Jit M, Hasso-Agopsowicz M, Henschke N, et al. Mortality reduction benefits and intussusception risks of rotavirus vaccination in 135 low-income and middle-income countries: a modelling analysis of current and alternative schedules. Lancet Glob Heal. 2019 Nov 1;7(11):e1541–52. https://doi.org/10.1016/S2214-109X(19)30412-7
**Age distribution of RVGE deaths**		
Log Logistic scale parameter	Beta-PERT (mid = best fit for Niger/U5MR stratum, range = 95% CI for Niger/U5MR stratum)	Hasso-Agopsowicz M, Ladva CN, Lopman B, Sanderson C, Cohen AL, Tate JE, et al. Global Review of the Age Distribution of Rotavirus Disease in Children Aged under 5 Years Before the Introduction of Rotavirus Vaccination. Clin Infect Dis. 2019 Jan 28. https://doi.org/10.1093/cid/ciz060
**Disability weights for calcualting DALYs**		
Percentage of healthy time lost whilst living with the disease		
Non Severe RVGE	Beta-PERT (mid = Salomon, GBD 2013, moderate diarhea, range = Salomon, GBD 2013, moderate diarrhea, 95% uncetainty interval)	Salomon JA, Haagsma JA, Davis A, Maertens De Noordhout C, Polinder S, Havelaar AH, et al. Disability weights for the Global Burden of Disease 2013 study. Vol. 3, Articles Lancet Glob Health. 2015. https://doi.org/10.1016/S2214-109X(15)00069-8
Severe RVGE	Beta-PERT (mid = Salomon, GBD 2013, severe diarhea, range = Salomon, GBD 2013, severe diarhea, 95% uncetainty interval)	
Non Severe RVGE	Beta-PERT (mid = ROSE trial, range = ROSE trial)	Isanaka S. et al. Rotavirus vaccine efficacy up to 2 years of age and against diverse circulating rotavirus strains in Niger: Extended follow-up of a randomized controlled trial. PLOS MEDICINE 2021 https://doi.org/10.1371/journal.pmed.1003655
Severe RVGE	Beta-PERT (mid = ROSE trial, range = ROSE trial)	
**Vaccine coverage**		
Doses with DTP1, DTP2, DTP3	Beta-PERT (mid = WUENIC 2020, range = WUENIC 2020 +/-10%)	WHO UNICEF coverage estimates WHO World Health Organization: Immunization, Vaccines And Biologicals. Vaccine preventable diseases Vaccines monitoring system 2020 Global Summary Reference Time Series: DTP3. Available from https://apps.who.int/immunization_monitoring/globalsummary/timeseries/tswucoveragedtp3.html Accessed on accessed 15 September 2021.
**Vaccine timeliness**		
Log Logistic scale parameter	Beta-PERT (mid = best fit for Niger or schedule stratum, range = median age +/-10%)	Clark A, Sanderson C. Timing of children's vaccinations in 45 low-income and middle-income countries: an analysis of survey data. Lancet. 2009;373(9674):1543–9. https://doi.org/10.1016/s0140-6736(09)60317-2
**Vaccine efficacy**		
Initial efficacy against RVGE mortality (2wks after dose administered)	Beta (alpha = 723·19, beta = 98·31, [A] = 0%, [B] = 100%)	Clark A, van Zandvoort K, Flasche S, Sanderson C, Bines J, Tate J, Parashar U, Jit M. Efficacy of live oral rotavirus vaccines by duration of follow-up: a meta-regression of randomised controlled trials. The Lancet Infectious Diseases. 2019;19(7):717–727. https://doi.org/10.1016/S1473-3099(19)30126-4
Mean duration of vaccine efficacy in months	Beta-PERT	
Relative efficacy of 1 dose versus 2/3 doses	Base value: 0·63 (0·51%-0·79%)<br>Beta (alpha = 27·05, beta = 15·68, [A] = 0, [B] = 1)	

For peer review only - http://bmjopen.bmj.com/site/about/guidelines.xhtml

Parameter	Probability distribution	Source
**Vaccine program costs and healthcare costs**	Beta-PERT (mid=ROSE costing studies, range = ROSE costing studies)	ROSE costing studies<br>Gavi, the Vaccine Alliance. Co-financing Policy. Version 2.0. Available from https://www.gavi.org/news/document-library/gavi-co-financing-policy Accessed on 9 September 2021.<br>Gavi, The Vaccine Alliance. Eligibility and Transition Policy. Version 3.0. Available from https://www.gavi.org/news/document-library/gavi-eligibility-and-transition-policy Accessed on 9 September 2021.<br>Baral R. et al. Cost of illness for childhood diarrhea in low- and middle-income countries: a systematic review of evidence and modelled estimates. BMC Public Health. 2020; 20: 619. https://dx.doi.org/10.1186%2Fs12889-020-08595-8

For peer review only

Table S4: Health and economic benefits of vaccine (2021-2030, discounted)

**Health and economic benefits**	**ROTARIX (2 doses)**	**ROTAVAC (3 doses)**	**ROTASIIL (3 doses)**
Non-severe RVGE cases averted	774,058	774,058	774,058
Non-severe RVGE visits averted	394,770	394,770	394,770
Severe RVGE cases averted	556,379	556,379	556,379
Severe RVGE visits averted	283,753	283,753	283,753
Severe RVGE hospitalisations averted	140,263	140,263	140,263
Deaths averted	21,631	21,631	21,631
Healthcare cost averted (US$)			
Government perspective	5,062,897	5,062,897	5,062,897
Societal perspective	7,714,859	7,714,859	7,714,859
DALYs averted	544,142	544,142	544,142
Vaccine programme cost (US$)	77,257,820	46,670,448	61,805,765
**Cost-effectiveness ratio (US$ per DALYs averted compared to no vaccine scenario)**			
Government perspective	133	76	104
Societal perspective	128	72	99

For peer review only

Figure S 1: Scenario analysis results

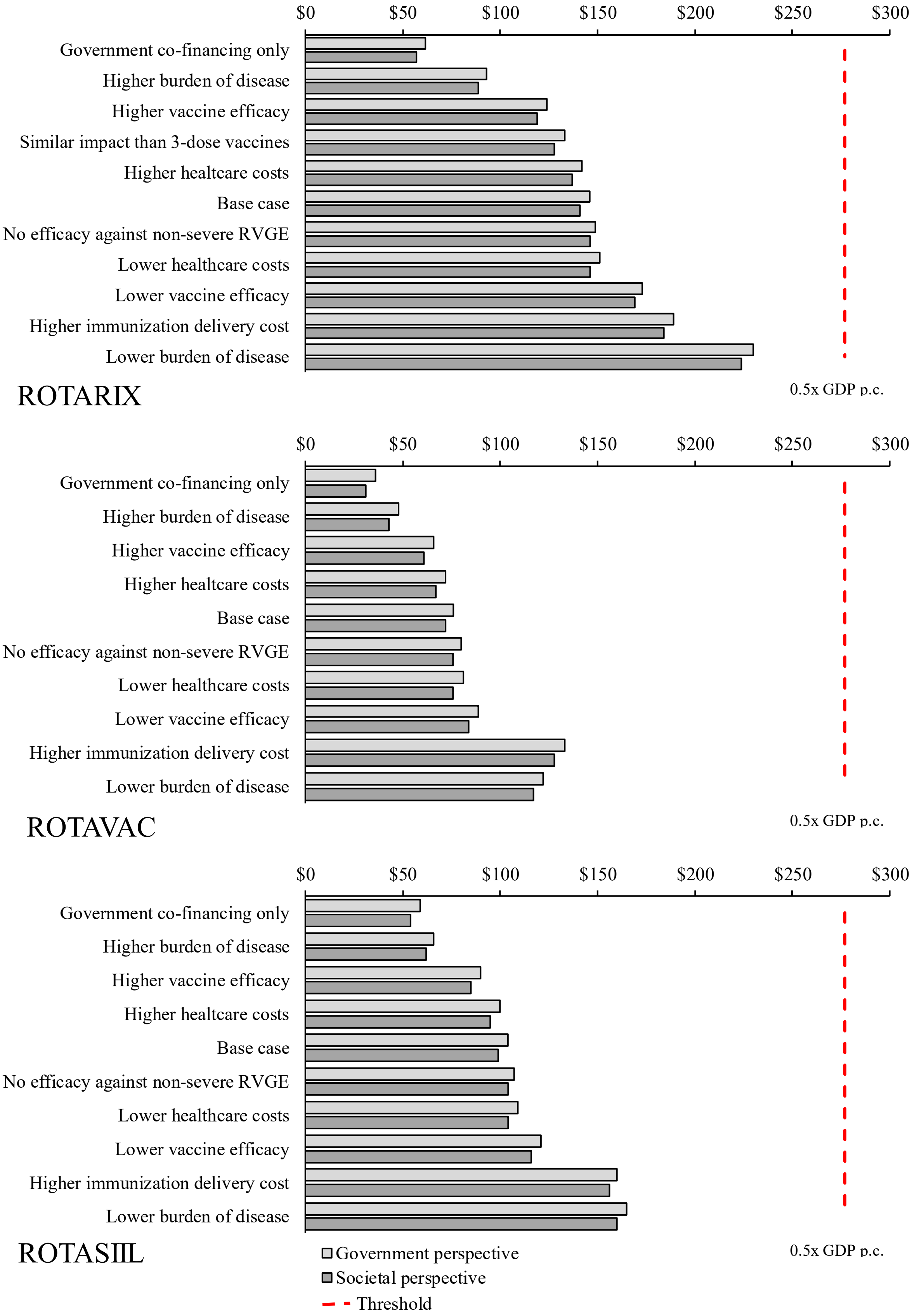

Consolidated Health Economic Evaluation Reporting Standards (CHEERS) 2022 Checklist

The CHEERS 2022 statement replaces the 2013 CHEERS statement, which should no longer be used. The CHEERS 2022 checklist contains 28 items with accompanying descriptions. Checklist users should indicate the section of the manuscript where relevant information can be found. The authors recommend using a section heading with a paragraph number. If an item does not apply to a particular economic evaluation, checklist users are encouraged to report "Not Applicable." If information is otherwise not reported, checklist users are encouraged to write, "Not Reported." Users should avoid the term "Not Conducted" as CHEERS is intended to guide and capture reporting. Additional information on CHEERS 2022 can be found here.

Title

1. Title

Identify the study as an economic evaluation and specify the interventions being compared.

Abstract

2. Abstract

Provide a structured summary that highlights context, key methods, results, and alternative analyses.

Introduction

3. Introduction: Background and Objectives

Give the context for the study, the study question, and its practical relevance for decision making in policy or practice.

Methods

4. Health economic analysis plan

© 2022 ISPOR—The Professional Society for Health Economics and Outcomes Research
International Society for Pharmacoeconomics and Outcomes Research, Inc

Indicate whether a health economic analysis plan was developed and where available.

5. **Study population**

Describe characteristics of the study population (such as age range, demographics, socioeconomic, or clinical characteristics).

6. **Setting and location**

Provide relevant contextual information that may influence findings.

7. **Comparators**

Describe the interventions or strategies being compared and why chosen.

8. **Perspective**

State the perspective(s) adopted by the study and why chosen.

9. **Time horizon**

State the time horizon for the study and why appropriate.

10. **Discount rate**

Report the discount rate(s) and reason chosen.

11. **Selection of outcomes**

Describe what outcomes were used as the measure(s) of benefit(s) and harm(s).

12. **Measurement of outcomes**

Describe how outcomes used to capture benefit(s) and harm(s) were measured.

© 2022 ISPOR—The Professional Society for Health Economics and Outcomes Research
International Society for Pharmacoeconomics and Outcomes Research, Inc.
For peer review only - http://bmjopen.bmj.com/site/about/guidelines.xhtml

13. Valuation of outcomes

Describe the population and methods used to measure and value outcomes.

____

14. Measurement and valuation of resources and costs

Describe how costs were valued.

____

15. Currency, price date, and conversion

Report the dates of the estimated resource quantities and unit costs, plus the currency and year of conversion.

---

16. Rationale and description of model

If modeling is used, describe in detail and why used. Report if the model is publicly available and where it can be accessed.

____

17. Analytics and assumptions

Describe any methods for analyzing or statistically transforming data, any extrapolation methods, and approaches for validating any model used.

____

18. Characterizing heterogeneity

Describe any methods used for estimating how the results of the study vary for subgroups.

____

19. Characterizing distributional effects

Describe how impacts are distributed across different individuals or adjustments made to reflect priority populations.

____

20. Characterizing uncertainty

Describe methods to characterize any sources of uncertainty in the analysis.

____ **21.**

21. Approach to engagement with patients and others affected by the study

© 2022 ISPOR—The Professional Society for Health Economics and Outcomes Research
International Society for Pharmacoeconomics and Outcomes Research, Inc
For peer review only - http://bmjopen.bmj.com/site/about/guidelines.xhtml

Describe any approaches to engage patients or service recipients, the general public, communities, or stakeholders (eg, clinicians or payers) in the design of the study.

____

Results

22. Study parameters

Report all analytic inputs (eg, values, ranges, references) including uncertainty or distributional assumptions.

____

23. Summary of main results

Report the mean values for the main categories of costs and outcomes of interest and summarize them in the most appropriate overall measure.

____

24. Effect of uncertainty

Describe how uncertainty about analytic judgments, inputs, or projections affects findings. Report the effect of choice of discount rate and time horizon, if applicable.

____

25. Effect of engagement with patients and others affected by the study

Report on any difference patient/service recipient, general public, community, or stakeholder involvement made to the approach or findings of the study.

____

Discussion

26. Study findings, limitations, generalizability, and current knowledge

Report key findings, limitations, ethical, or equity considerations not captured and how these could impact patients, policy, or practice.

____

© 2022 ISPOR—The Professional Society for Health Economics and Outcomes Research
International Society for Pharmacoeconomics and Outcomes Research, Inc
For peer review only - http://bmjopen.bmj.com/site/about/guidelines.xhtml

Other Relevant Information

27. Source of funding

Describe how the study was funded and any role of the funder in the identification, design, conduct, and reporting of the analysis.

---

28. Conflicts of interest

Report authors' conflicts of interest according to journal or International Committee of Medical Journal Editors requirements.

© 2022 ISPOR—The Professional Society for Health Economics and Outcomes Research
International Society for Pharmacoeconomics and Outcomes Research, Inc.
For peer review only - http://bmjopen.bmj.com/site/about/guidelines.xhtml

BMJ Open

Impact and cost-effectiveness of rotavirus vaccination in Niger: a modeling study evaluating alternative rotavirus vaccines

Journal:	*BMJ Open*
Manuscript ID	bmjopen-2022-061673.R1
Article Type:	Original research
Date Submitted by the Author:	30-Jun-2022
Complete List of Authors:	Debellut, Frédéric ; PATH<br>Tang, Kevin; Epicentre; London School of Hygiene & Tropical Medicine<br>Clark, Andrew; London School of Hygiene and Tropical Medicine, Health Services Research and Policy<br>Pecenka, Clint; PATH<br>Assao, Bachir; Epicentre<br>Guindo, Ousmane; Epicentre<br>Grais, RF; Epicentre<br>Isanaka, Sheila; Epicentre; Harvard TH Chan School of Public Health, Departments of Nutrition and Global Health and Population
**Primary Subject Heading**:	Health economics
Secondary Subject Heading:	Health policy, Public health
Keywords:	Health economics < HEALTH SERVICES ADMINISTRATION & MANAGEMENT, Health policy < HEALTH SERVICES ADMINISTRATION & MANAGEMENT, Public health < INFECTIOUS DISEASES, Paediatric infectious disease & immunisation < PAEDIATRICS

SCHOLARONE™
Manuscripts

I, the Submitting Author has the right to grant and does grant on behalf of all authors of the Work (as defined in the below author licence), an exclusive licence and/or a non-exclusive licence for contributions from authors who are: i) UK Crown employees; ii) where BMJ has agreed a CC-BY licence shall apply, and/or iii) in accordance with the terms applicable for US Federal Government officers or employees acting as part of their official duties; on a worldwide, perpetual, irrevocable, royalty-free basis to BMJ Publishing Group Ltd ("BMJ") its licensees and where the relevant Journal is co-owned by BMJ to the co-owners of the Journal, to publish the Work in this journal and any other BMJ products and to exploit all rights, as set out in our licence.

The Submitting Author accepts and understands that any supply made under these terms is made by BMJ to the Submitting Author unless you are acting as an employee on behalf of your employer or a postgraduate student of an affiliated institution which is paying any applicable article publishing charge ("APC") for Open Access articles. Where the Submitting Author wishes to make the Work available on an Open Access basis (and intends to pay the relevant APC), the terms of reuse of such Open Access shall be governed by a Creative Commons licence – details of these licences and which Creative Commons licence will apply to this Work are set out in our licence referred to above.

Other than as permitted in any relevant BMJ Author's Self Archiving Policies, I confirm this Work has not been accepted for publication elsewhere, is not being considered for publication elsewhere and does not duplicate material already published. I confirm all authors consent to publication of this Work and authorise the granting of this licence.

For peer review only

Impact and cost-effectiveness of rotavirus vaccination in Niger: a modeling study evaluating alternative rotavirus vaccines

Authors

Frédéric Debellut[1], Kevin Tang[2,3], Andrew Clark[2], Clint Pecenka[4], Bachir Assao[5], Ousmane Guindo[5], Rebecca F. Grais[3], Sheila Isanaka[3,6]

1. Center for Vaccine Innovation and Access, PATH, Rue de Varembé 7, 1202 Geneva, Switzerland.

2. London School of Hygiene and Tropical Medicine, Keppel Street, London WC1E 7HT, United Kingdom.

3. Epicentre, Paris, France

4. Center for Vaccine Innovation and Access, PATH, 2201 Westlake Ave, Suite 200, Seattle, WA 98121, USA.

5. Epicentre, Niamey, Niger

6. Departments of Nutrition and Global Health and Population, Harvard T.H. Chan School of Public Health, 655 Huntington Avenue, Boston, MA, 02115 USA

Corresponding author: Frédéric Debellut fdebellut@path.org

For peer review only

Abstract

Objectives: To evaluate the cost-effectiveness of alternative rotavirus vaccines in Niger, using UNIVAC, a proportionate outcomes model.

Setting: The study leverages global, regional and local data to inform cost-effectiveness modelling. Local data were collected as part of a clinical trial taking place in the Madarounfa district, Maradi region, Niger.

Participants: The study models impact of infants vaccination on rotavirus gastroenteritis in children under five years of age.

Interventions: We compared the use of ROTARIX, ROTAVAC (Bharat Biotech, India), and ROTASIIL (Serum Institute, India) to no vaccination and to each other over a ten-year period starting in 2021.

Results: We estimated that ROTARIX, ROTAVAC, and ROTASIIL would each prevent 13 million cases and 20,000 deaths of children under five years over a ten-year period in Niger. Compared to no vaccination, the cost to avert a DALY was $146 with ROTARIX, $107 with ROTASIIL, and $76 with ROTAVAC from the government perspective. ROTAVAC dominated ROTARIX and ROTASIIL (e.g., provided similar or higher benefits at a lower cost) and had 90 percent chance to be cost-effective at a $100 willingness-to-pay threshold.

Conclusions: This study can inform decision-making around rotavirus vaccination policy in Niger, demonstrating that ROTAVAC is likely the most cost-effective option. Alternative products (ROTASIIL and ROTARIX) may also be considered by decision makers if they are priced more competitively, or if their cold chain requirements could bring additional economic benefits.

For peer review only

Strengths and limitations of this study

- This analysis uses a cost-effectiveness model (UNIVAC) developed to assist decision-makers in low- and middle-income countries in evaluating vaccine policy options.
- This economic evaluation leverages unique local data on cost to deliver rotavirus vaccines and cost borne by households for their children sick with rotavirus gastroenteritis.
- Local data was collected in a single region of the country, and sensitivity analysis was performed to handle uncertainty around their representativeness for Niger.

For peer review only

Introduction

Rotavirus remains the leading cause of diarrhea mortality globally and was estimated to cause more than 150,000 deaths in children younger than five years of age in 2019, with more than 80% of these deaths occurring in sub-Saharan Africa.[1,2] The World Health Organization (WHO) recommends universal rotavirus vaccination for all children.[3]

Two live oral rotavirus vaccines (ROTARIX® [GlaxoSmithKline, Belgium] and RotaTeq® [Merck & Co., Inc., United States]) were prequalified and approved for global use by WHO in 2009.[4] One decade later, in 2018, two additional live oral rotavirus vaccines (ROTAVAC® [Bharat Biotech, India] and ROTASIIL® [Serum Institute, India]) entered the global market and received the same WHO prequalification status. These rotavirus vaccines are safe and effective and are used in more than 110 countries worldwide.[5,6]

In many of the world's poorest countries, including several African countries, rotavirus vaccines have been introduced with financial support from Gavi, the Vaccine Alliance. Gavi is an international organization aiming to increase equitable and sustainable access to vaccines for low- and middle-income countries. As a result, countries eligible for Gavi support can access negotiated prices from manufacturers through procurement with UNICEF. In 2014, the Republic of the Niger (hereafter referred to as Niger) introduced ROTARIX into its national immunization program with support from Gavi.[7] Prior to this, rotavirus was associated with around one-third of all severe diarrhea cases with moderate-to-severe dehydration in children aged under five years in the country.[8] The ROtavirus Safety and Efficacy (ROSE) clinical trial, implemented by Epicentre along with the Niger Ministry of Health and Médecins Sans Frontières, demonstrated the safety and efficacy of ROTASIIL in areas of Niger where ROTARIX was not yet available.[9,10] The ROSE clinical trial also collected data to estimate the cost of delivering rotavirus vaccine as part of the national immunization program as well as the costs borne by households when a child develops rotavirus illness.[10]

For peer review only - http://bmjopen.bmj.com/site/about/guidelines.xhtml

Despite inclusion of ROTARIX in the Expanded Programme on Immunization (EPI) program for several years and the availability of alternative rotavirus vaccines, there has been no formal economic evaluation of any rotavirus vaccine in Niger. This study aims to evaluate and compare the cost-effectiveness of ROTARIX, ROTAVAC, and ROTASIIL using locally collected data. The results of this study can inform national decision-making on rotavirus vaccination policy in Niger.

Methods

Study design

We estimated the health impact and cost-effectiveness of three different rotavirus vaccines in Niger from 2021 to 2030 from the government and societal perspectives. The government perspective includes healthcare costs supported by the government only, while the societal perspective also accounts for healthcare costs supported by households. We used an Excel proportionate outcomes model, UNIVAC (version 1.4.30), to compare three rotavirus vaccine products to no vaccination and to each other.[11] We evaluated the three rotavirus vaccines available through Gavi: ROTARIX, a monovalent human liquid vaccine available in a single-dose vial presentation and administered in a two-dose course; ROTAVAC, a monovalent human-bovine reassortant frozen liquid vaccine, available in a 5-dose vial presentation and administered in a three-dose course; and ROTASIIL, a pentavalent human-bovine reassortant lyophilized vaccine available in a 2-dose vial presentation and administered in a three-dose course.3 We did not evaluate ROTATEQ as it is not available to Gavi-eligible countries and therefore would be unlikely considered for use in Niger by governmental authorities.[12]

The UNIVAC model was used to predict the number of averted rotavirus disease events in children under five years of age, related healthcare costs, and vaccination program costs. Input parameters were informed by data from the literature and primary data collected during the ROSE trial and its ancillary costing studies. The primary outcome measure is the incremental cost-effectiveness ratio (ICER) expressed as the cost in US$ per disability-adjusted life-year (DALY) averted. In the absence of a set willingness-to-pay

(WTP) threshold in Niger, we interpret our cost-effectiveness results using 0.5 times the GDP per capita (Niger's GDP per capita was US$553.9 in 2019[13]). All cost data are reported in 2021 USD, and we applied a standard 3% discount rate to all future costs and health outcomes.

Parent trial and ancillary costing studies

The ROSE trial was a double-blind, placebo-controlled randomized phase III trial that was implemented in the Madarounfa district in Niger from August 2014 to February 2018 (ClinicalTrials.gov identifier: NCT02145000). It assessed the efficacy and safety of ROTASIIL vaccine administered at 6, 10, and 14 weeks of age against rotavirus disease in healthy infants, demonstrating the safety of the vaccine and an efficacy level of 54.7% (95% CI 38.1% to 66.8%) at 24 months of follow-up.[10,14] Two ancillary costing studies were carried out alongside the trial to collect data on 1) the costs of immunization delivery in the national immunization program and 2) the costs of illness borne by households when they seek care for their child sick with rotavirus diarrhea.

For costs of immunization delivery, we included cost categories according to standard guidelines developed for the EPIC studies.[15] Primary data was collected from the central EPI at the national, regional, and district levels, as well as at four health facilities. Data collected covered international handling and transportation costs, national, regional, and district costs related to storing and distributing vaccines, the number of doses of vaccines stored and distributed, and the vaccines' presentation. This data was used to define a volume-based unit cost, which was then applied to each vaccine and their respective volume. We added costs at the health facility level, which was composed of labour costs to administer vaccines, storage, and transportation costs. Details on costs included for immunization delivery are available from supplementary Table S1. The main driver of cost was service delivery at health facility level, which represented between 30% and 60% of the total immunization delivery cost, depending on which vaccine was considered.

For peer review only

Healthcare costs of rotavirus gastroenteritis (RVGE) illness are estimated from primary data collected within the ROSE trial as well as published estimates. For the government perspective, we used estimates of direct medical costs of diarrhea as published by Baral *et al*.[16] For the societal perspective, direct medical costs were combined with household costs collected from a sub-group of caregivers presenting to ROSE trial health facilities with a child sick with RVGE.[17] Household costs included out-of-pocket expenditures to seek care in terms of medicines, transportation, and food as well as indirect costs from time spent seeking care and caring for sick children. Caregiver time was valued using the annual GDP per capita divided by 365 days and multiplied by the number of days spent seeking care or caring for a child in hospital. Data were collected from February 2018 to April 2019 from caregivers presenting to an outpatient health facility (n = 365) or whose child has been hospitalized (n = 26). Data was collected using standard questionnaires used to gather information on cost incurred by families during an episode.

All cost categories and line items included during this ancillary costing activities are available from supplementary Table S2.

Rotavirus disease burden

We assumed that rotavirus infection would manifest as either non-severe RVGE or severe RVGE, aligning with classifications used in the trial. Non-severe RVGE resolves after seeking care in an outpatient setting or without seeking any care. Severe RVGE may resolve or be fatal, after seeking care in an outpatient or an inpatient setting or without seeking any care. Input parameters used for modeling rotavirus disease burden in Niger are available in Table 1. To define incidence rates for the different disease events, we used the incidence of severe and non-severe RVGE from the ROSE trial placebo population (6 weeks to 2 years of age) and converted this to incidence among children aged <5 years using data on the age distribution of rotavirus hospital admissions in high-mortality settings[18]. We defined incidence of RVGE clinic visits using the proportion of mothers reporting seeking care for their child sick with diarrhea (51%) based the most recently published DHS survey in Niger.[19] We used ROSE trial data to inform the incidence of severe RVGE hospitalizations, using the proportion of children with severe

For peer review only

142 RVGE that were brought to the hospital (25%). We also used the ROSE trial data to inform the duration of severe and non-severe RVGE episodes. Lower and upper bounds for incidence reflect the low and high estimates used for Niger in previously published multi-country analyses.[20,21] The rate of RVGE deaths was taken from Clark *et al* (2019)[22] and represents the mean of three possible international estimates of RVGE mortality in Niger. DALY weights were taken from the Global Burden of Disease (GBD) study, using moderate and severe diarrhea as proxies for non-severe and severe RVGE.[23]

For peer review only

Table 1: Input parameters for estimation of disease burden

Input parameters	Base case	Lower bound	Upper bound	References
**Incidence (per 100,000 under-five children per year)**				
Non-severe RVGE cases	3,260	2,305	4,701	[10,18]
Non-severe RVGE visits	1,662	1,176	2,398	[19]
Severe RVGE cases	1,992	1,354	2,550	[10,18]
Severe RVGE visits	1,016	690	1,301	[19]
RVGE Hospitalisations	502	341	643	[10]
RVGE Deaths	115	74	176	[22]
**Duration of illness (days)**				
Non-severe RVGE case	2.36	2.13	2.59	[10]
Severe RVGE cases	3.58	3.07	4.09	[10]
**Disability weights**				
Non-severe RVGE case	0.188	0.125	0.264	[23]
Severe RVGE cases	0.247	0.164	0.348	
**Age distribution of disease events**				
**Age distribution**	**Cumulative percentage**			
< 1 month	0%			[18]
< 2 months	3%			
< 3 months	10%			
< 6 months	36%			
< 1 year	80%			
< 2 years	97%			
< 3 years	99%			
< 4 years	100%			
< 5 years	100%			

For peer review only

Vaccine efficacy, coverage, and coverage timeliness

Efficacy data for the three evaluated vaccines is taken from a meta-analysis pooling data from all published randomized controlled trials.[24] We assume that all products confer the same level of protection after the last dose of a full course, corresponding to 79% efficacy two weeks after the last dose, with subsequent waning bringing efficacy to 45% 12 months after the last dose.

We assume that vaccine efficacy against non-severe RVGE was 85% of the efficacy assumed for severe RVGE.[25] In an alternative scenario, we assumed 0% vaccine efficacy against non-severe disease, as can be derived from the ROSE trial, when comparing the reported efficacy against severe RVGE and efficacy against RVGE of any severity.[10]

Vaccine coverage and timeliness data for rotavirus vaccine doses 1, 2, and 3 were assumed to be the same as vaccine coverage and timeliness reported for diphtheria, tetanus, and pertussis (DTP) vaccine, where three doses of the vaccine are scheduled to be administered at 6, 10, and 14 weeks of age. For 2-dose ROTARIX, we only applied coverage for DTP1 and DTP2. Vaccine timeliness data were based on analyses by Clark *et al*.[26,22,27]

Vaccine prices and other procurement costs

We used vaccine prices published by Gavi, the Vaccine Alliance and assumed such prices would be available for the entire period of analysis.[28] In addition to benefitting from Gavi-negotiated prices, eligible countries only pay a share of vaccine procurement, dependent on their income per capita. In a scenario analysis, we applied the Niger co-financing share as an initial self-financing country per Gavi policy.[29,30] Other vaccine procurement related costs include safety boxes and vaccine wastage.

Vaccine wastage accounts for vaccine doses that are discarded, lost, damaged, or destroyed. Wastage is dependent on each vaccine presentation. We used wastage rates as recommended by Gavi's detailed product profiles and calculated with the WHO wastage rate tool. This resulted in a 4% wastage rate for

For peer review only

ROTARIX, 5% for ROTAVAC, and 9% for ROTASIIL.[28,31] All Input parameters used for estimation of vaccine program costs and health care costs are available from Table 2.

For peer review only

Table 2: Input parameters for estimation of vaccine program costs and health care costs

Input parameter	Base case	Lower bound	Upper bound	Sources
**Vaccine coverage***				
Dose 1	92%	82.8%	100%	[26]
Dose 2	86.5%	77.9%	95.2%	
Dose 3 (ROTAVAC and ROTASIIL only)	81%	72.9%	89.1%	
**Vaccine price per dose (US$)**				
ROTARIX	$2.33	$0.20	-	[28]
ROTAVAC	$0.85	$0.13	-	
ROTASIIL	$0.95	$0.13	-	
**% Wastage**				
ROTARIX	4%	2%	6%	[28]
ROTAVAC	10%	5%	15%	
ROTASIIL	9%	5%	15%	
**Immunization delivery cost per dose (2021 US$)**				
ROTARIX	$1.65	$1	$2.77	ROSE costing study
ROTAVAC	$0.75	$0.75	$1.87	
ROTASIIL	$1.20	$1	$2.31	
**Health care cost (2021US$)**				
Unit cost of RVGE visit<br>Government perspective	$4.79	$2.40	$7.19	[16] and ROSE costing study for households' costs
Societal perspective	$7.16	$4.77	$9.56	
Unit cost of severe RVGE hospitalisation<br>Government perspective	$18.38	$9.19	$27.57	
Societal perspective	$28.68	$19.49	$37.87	

* For vaccine timeliness we assume that by 6 months of age, 91% of children would have received their first dose, 80% would have received their second dose, and, when applicable, 60% would have received their third dose of rotavirus vaccine. Data were fitted using a gamma curve to estimate timeliness by week of age <5 years.

For peer review only

Sensitivity analyses

To account for uncertainty, we undertook deterministic and probabilistic uncertainty analyses. Deterministic analyses covered a series of scenarios more and less favorable to the vaccine. In scenario 1, we accounted only for the country co-financing Niger currently pays for vaccine procurement; in scenario 2, we accounted for a higher burden of disease; in scenario 3, we accounted for a lower burden of disease; in scenario 4, we accounted for a higher vaccine efficacy; in scenario 5, we assumed a lower vaccine efficacy; in scenario 6, we accounted for higher healthcare costs; in scenario 7, for lower healthcare costs; in scenario 8, for higher immunization delivery costs; and in scenario 9, we assumed vaccines had no efficacy against non-severe RVGE. Finally, as our assumptions around similar efficacy of the last dose of vaccine led to lower impact of the 2-dose ROTARIX vaccine compared to 3-dose ROTAVAC and ROTASIIL vaccines, we also evaluated the cost-effectiveness of ROTARIX with similar impact to the 3-dose vaccines (scenario 10).

We ran a probabilistic uncertainty analysis for the three vaccines. We conducted 1,000 model runs sampling input data and statistical distributions available in supplementary table S3. We reported, for each vaccine, the mean ICER value from the government perspective as well as results for each model run. We also built a cost-effectiveness acceptability curve showing the probability for the dominant vaccine to be cost-effective at different WTP thresholds.

No Patient and Public Involvement

This research was done without patient or public involvement. It was not appropriate or possible to involve patients or the public in the design, or conduct, or reporting, or dissemination of our research as the study focused on assessing cost and cost-effectiveness of an intervention provided by the Ministry of Health.

For peer review only

207 **Results**

208 *Deterministic analysis*

209 We estimate that a vaccination program using any of the three rotavirus vaccines analysed could avert
210 around 13 million RVGE cases and 20,000 deaths over 10 years in Niger. With 660,000 visits and
211 136,000 hospitalizations averted, rotavirus vaccination could result in healthcare cost savings of US$5
212 million from the government and US$7 million from the societal perspective. Vaccination program costs
213 vary depending on which vaccine is used: US$46.7 million with ROTAVAC, US$61.8 million with
214 ROTASIIL, and US$77.3 with ROTARIX, when not accounting for Gavi subsidies for vaccine
215 procurement. Detailed figures on outcomes related to each vaccine are available in Table 3. On average,
216 undiscounted annual vaccination program costs in absence of Gavi support vary between US$5.5 million
217 with ROTAVAC and US$8.9 million with ROTARIX, with the majority of the cost dedicated to vaccines
218 and supplies procurement (55% for ROTAVAC, 60% with ROTARIX).

219 The cost per DALY averted varies by vaccine and perspective. From the government perspective, and
220 when comparing each vaccine to 'no vaccine', the most favourable ICER is with ROTAVAC (US$76 per
221 DALY averted), followed by ROTASIIL (US$104) and then ROTARIX (US$146). From the societal
222 perspective, the same order remains, with ROTAVAC having the lowest ICER (US$72), followed by
223 ROTASIIL (US$99) and ROTARIX (US$141). When assuming 2-dose ROTARIX had similar impact to
224 the 3-dose ROTAVAC or ROTASIIL, the ROTARIX ICER decreased to $133 from the government
225 perspective and $128 from the societal perspective (Table S4).

226 ROTAVAC can be considered cost-effective based on a WTP threshold set at half the GDP per capita
227 (US$277). Both ROTARIX and ROTASIIL may not be cost-effective given the availability of
228 ROTAVAC; i.e., if ROTARIX is compared directly to ROTAVAC, the cost per DALY averted would be
229 US$638, and if ROTASIIL is compared directly to ROTAVAC, health benefits are the same, but
230 vaccination program costs are higher (ICER is undefined). However, there is considerable uncertainty

For peer review only

231 around several of our inputs and the rank order would change if there were changes in important

232 variables.

233

For peer review only

Table 3: Health and economic benefits of vaccine (2021-2030, discounted)

**Health and economic benefits**	**ROTARIX (2 doses)**	**ROTAVAC (3 doses)**	**ROTASIIL (3 doses)**
Non-severe RVGE cases averted	706,843	774,058	774,058
Non-severe RVGE visits averted	360,490	394,770	394,770
Severe RVGE cases averted	508,066	556,379	556,379
Severe RVGE visits averted	259,113	283,753	283,753
Severe RVGE hospitalisations averted	128,083	140,263	140,263
Deaths averted	19,752	21,631	21,631
Healthcare cost averted (US$)			
Government perspective	4,623,393	5,062,897	5,062,897
Societal perspective	7,045,141	7,714,859	7,714,859
DALYs averted	496,905	544,142	544,142
Vaccine programme cost (US$)	77,257,820	46,670,448	61,805,765
**Cost-effectiveness ratio (US$ per DALYs averted compared to no vaccine scenario)**			
Government perspective	146	76	104
Societal perspective	141	72	99

Scenario analysis

The scenario analysis shows that all three rotavirus vaccines have cost-effectiveness ratios below the half GDP per capita threshold (Figure S1). The most favourable scenario is the scenario where we account for the level of co-financing currently supported by the country. When accounting for vaccine co-financing instead of the Gavi price from the government perspective, ROTAVAC has a cost-effectiveness ratio of US$36, ROTASIIL US$59, and ROTARIX US$62. The least favourable scenario is the scenario where we account for a lower burden of disease. In this scenario, from the government perspective, the cost to avert a DALY is US$122 with ROTAVAC, US$165 with ROTASIIL, and US$230 with ROTARIX.

Probabilistic sensitivity analysis

For peer review only

245 Cost-effectiveness of rotavirus vaccinination in Niger is further confirmed by the probabilistic uncertainty analysis whereby all 1,000 runs returned cost-effectiveness ratio values below the assumed threshold. The same order of the vaccines' cost-effectiveness ratios remains, with ROTAVAC dominating other options (less costly and better health outcome) at a mean ICER of US$73 per DALY averted, ROTASIIL at US$103, and ROTARIX at US$132 (Figure 1). The cost-effectiveness acceptability curve shows the high probablity for the dominant vaccine option to be cost-effective at thresholds lower than $277. ROTAVAC has >99% chance of being cost-effective at a WTP threshold of $150 (Figure 2).

For peer review only

Discussion

Rotarix was officially included in the national immunization program in Niger in 2014, but no economic evaluation was conducted at the time nor widespread use. In 2014 only two vaccine presentations were available (ROTARIX and RotaTeq). At end of 2021, there are more than 10 rotavirus vaccine presentations that countries can choose from, making continued analysis essential to make informed choices.

This analysis confirms that rotavirus vaccine averts a substantial burden in Niger and remains cost-effective several years after introduction. All vaccines evaluated have ICERs below half of the GDP per capita WTP threshold, despite each product's differences in presentation and characteristics. Cost-effectiveness ratios for the vaccines are generally of similar scale, with ROTAVAC dominating other options. ROTAVAC's economic advantage is mainly driven by its lower volume resulting in lower immunization delivery cost and lower price. This finding is consistent across all scenarios and probabilistic analyses and remains true even in the current context with Niger paying a share of the vaccine price through Gavi co-procurement. This study demonstrates that product presentations and their associated costs should be evaluated closely.

There are limits to this analysis worth highlighting. The immunization cost of delivery values used in the study are calculated based on the volume of each product. This advantages the product with the lowest cold chain footprint. Other factors should be considered when evaluating or revaluating product selection. In our example, policymakers may wish to consider the feasibility of using a product with a smaller cold chain footprint that also must be stored in the negative cold chain at central level. ROTASIIL, though a higher volume product, could be, under certain circumstances, stored outside of the cold chain. If use of such a thermostable product was feasible country-wide in Niger without incurring additional costs, then ROTASIIL would potentially become the best choice from an economic perspective. Additional costs may be incurred from having to develop separate processes for an individual vaccine. Experiences to date with use of vaccines outside of the cold chain, through the Controlled Temperature Chain (CTC) protocol,

For peer review only

have led to positive outcomes in campaign settings. This approach is, however, not currently recommended for routine vaccines.[32]

The ROSE trial provided unique primary data on cost of delivery and households' cost of illness. Immunization cost of delivery data are coming from a limited number of facilities, from one region only. While we address this limitation through sensitivity analysis, the immunization delivery cost data would benefit from being based on a higher set of facilities and additional regions and districts. The same limitation applies to the cost of illness data used in the modelling. The small sample for hospitalized children was collected from a single facility within the ROSE trial.

Different impact values with 2-dose versus 3-dose vaccines are based on how the model calculates health outcomes based on efficacy values and waning of protection after the last dose of vaccine. The third dose of ROTAVAC and ROTASIIL is given 4 weeks later and thus translates in a small delay in the waning of protection compared with ROTARIX. This in turns results in a slightly higher number of averted cases, visits, hospitalizations, and deaths. This has not been proven empirically, and studies that have assessed the impact of additional doses of vaccines have reported inconsistent results.[33] However, this is unlikely to impact our findings as, when accounting for similar impact among the three vaccines, the ROTARIX ICER remains inferior of that of ROTAVAC or ROTASIIL ($133 per DALY averted from the government perspective). The limited data available on large-scale ROTAVAC and ROTASIIL effectiveness and safety data on these two vaccines, are two other factors that represent limits to our findings. A recent impact study in Palestine showed that benefits of rotavirus vaccination were sustained two years after a switch from ROTARIX to ROTAVAC.[34]

This evaluation does not account for indirect effects of vaccination. However, considering recent work by *Ferrari et al*., it looks like indirect effects may not be a key driver in Niger and that our static model may provide reasonable results.[35]

For peer review only

Despite the involvement of national authorities and stakeholders, and use of locally collected data, there may be additional factors influencing decision-making that are not accounted for in the analysis.

The findings of this study may nevertheless contribute to informing decision-making around rotavirus vaccination policy in Niger, demonstrating that ROTAVAC is likely to be the most cost-effective option. Alternative products (ROTASIIL and ROTARIX) may also be considered by decision makers if they can be priced more competitively, or if their cold chain requirements could bring additional economic benefits. Regular re-evaluation is likely to be required as more evidence emerges on the relative costs and benefits of each product. Emerging data on the cost to switch vaccines should also be considered as a factor to account for when making decision about a change in product. Findings from few countries that have switched rotavirus vaccines in the past years are available and additional data is expected to become available with a number of countries planning to switch vaccines in 2022.[36,37]

Figure 1: Mean ICER and probabilistic cost-effectiveness results from the probabilistic uncertainty analysis, government perspective, over a 10-year period

Figure 2: Cost-effectiveness acceptability curve for the dominant option (ROTAVAC)

For peer review only

Data availability statement

The authors confirm that the data supporting the findings of this study are available within the article and its supplementary materials. The model used is available online at https://www.paho.org/en/provac-toolkit

ROSE trial data: The deidentified dataset supporting this research can be made available following a submitted request as per Epicentre and General Data Protection Regulation (EU) 2016/679 data sharing policy. Additional information is available at https://epicentre.msf.org/en/data-access-request.

Funding statement

This work was supported, in whole or in part, by the Bill & Melinda Gates Foundation [OPP1147721]. Under the grant conditions of the Foundation, a Creative Commons Attribution 4.0 Generic License has already been assigned to the Author Accepted Manuscript version that might arise from this submission.

ROSE trial funding: Funding was provided to Epicentre by Médecins Sans Frontières (MSF)-Operational Center Geneva (https://www.msf.org/) and the Kavli Foundation, Norway (https://www.kavlifoundation.org/). Serum Institute of India Pvt Limited provided vaccine and placebo in kind.

Ethics approval statement

Ethical assessment was undertaken, and approval given, by the Comité National d'Ethique pour la Recherche en Santé, CNERS, Ministère de la Santé Publique, République du Niger. Délibération N°28/2018/CNERS.

Contributorship statement

FD, KT, CP and SI designed the study. FD, KT, BA and OG collected and analyzed the data. AC developed the study model. CP, AC, RG, SI provided technical inputs to the study. FD ran the analysis and wrote the first draft of the manuscript. All authors reviewed and approved the final manuscript.

For peer review only

342 **Conflict of interest statement**

343 The authors have no conflict to report.

For peer review only

344 **References**

[1] Troeger C, Forouzanfar M, Rao PC, Khalil I, Brown A, Reiner RC, Fullman N, Thompson RL, Abajobir A, Ahmed M, et al. Estimates of global, regional, and national morbidity, mortality, and aetiologies of diarrhoeal diseases: a systematic analysis for the Global Burden of Disease Study 2015. The Lancet Infectious Diseases. 2017;17(9):909–948. doi:10.1016/S1473-3099(17)30276-1

[2] GBD Results Tool. Institute for Health Metrics and Evaluation. Available from http://ghdx.healthdata.org/gbd-results-tool Accessed on 7 April 2021.

[3] WHO. Rotavirus vaccines: WHO position paper – July 2021. Weekly Epidemiological Record, N°28, 16 July 2021. Available from https://apps.who.int/iris/handle/10665/346571 Accessed on 20 October 2019.

[4] WHO. Summary of Key Characteristics of WHO Prequalified Rotavirus Vaccines. WHO/IVB/2021.03. Available from file:///C:/Users/fdebellut/Downloads/WHO-IVB-2021.03-eng%20(1).pdf Accessed on 21 October 2021.

[5] Bergman H, Henschke N, Hungerford D, Pitan F, Ndwandwe D, Cunliffe N, Soares-Weiser K. Vaccines for preventing rotavirus diarrhoea: vaccines in use. Cochrane Database of Systematic Reviews 2021, Issue 11. Art. No.: CD008521. https://doi.org/10.1002/14651858.CD008521.pub6

[6] ViewHub. International Vaccine Access Center (IVAC). Available from https://view-hub.org/ Accessed on 25 March 2021.

[7] JSI Research and Training Institute Inc. Lessons Learned: Rotavirus & PCV Vaccine Introduction in Niger. March 2016. Available from https://publications.jsi.com/JSIInternet/Inc/Common/_download_pub.cfm?id=17995&lid=3 Accessed on 21 October 2021.

[8] Page A., Jusot V., Mamaty A., et al. Rotavirus Surveillance in Urban and Rural Areas of Niger, April 2010–March 2012. Emerging Infectious Diseases. 2014 Apr; 20(4): 573–580. https://dx.doi.org/10.3201%2Feid2004.131328

[9] Isanaka S., Guindo O., Langendorf C., et al. Efficacy of a Low-Cost, Heat-Stable Oral Rotavirus Vaccine in Niger. New England Journal of Medicine 2017; 376:1121-1130

[10] Isanaka S. et al. Rotavirus vaccine efficacy up to 2 years of age and against diverse circulating rotavirus strains in Niger: Extended follow-up of a randomized controlled trial. PLOS MEDICINE 2021 https://doi.org/10.1371/journal.pmed.1003655

[11] PAHO. ProVac Toolkit. Tools. About UNIVAC. Available from https://www.paho.org/en/provac-toolkit Accessed on 03 December 2021.

[12] NPR. Merck Pulls Out Of Agreement To Supply Life-Saving Vaccine To Millions Of Kids. November 1, 2018. Available from https://www.npr.org/sections/goatsandsoda/2018/11/01/655844287/merck-pulls-out-of-agreement-to-supply-life-saving-vaccine-to-millions-of-kids?t=1638525408118 Accessed on 03 December 2021.

[13] World Bank national accounts data, and OECD National Accounts data files. GDP per capita (current US$) – Niger. Available from https://data.worldbank.org/indicator/NY.GDP.PCAP.CD?locations=NE Accessed on 1 September 2021.

[14] Coldiron M., Guindo O., Makarimi R., et al. Safety of a heat-stable rotavirus vaccine among children in Niger: Data from a phase 3, randomized, double-blind, placebo-controlled trial. Vaccine. Volume 36, Issue 25, 14 June 2018, Pages 3674-3680. https://doi.org/10.1016/j.vaccine.2018.05.023

For peer review only

[15] Brenzel L., Young D., Walker D.G. Costs and financing of routine immunization: Approach and selected findings of a multi-country study (EPIC). Vaccine.2015 May 7;33 Suppl 1:A13-20. https://doi.org/10.1016/j.vaccine.2014.12.066

[16] Baral R. et al. Cost of illness for childhood diarrhea in low- and middle-income countries: a systematic review of evidence and modelled estimates. BMC Public Health. 2020; 20: 619. https://dx.doi.org/10.1186%2Fs12889-020-08595-8

[17] World Health Organization. (2005). Guidelines for estimating the economic burden of diarrhoeal disease, with focus on assessing the costs of rotavirus diarrhoea. World Health Organization. https://apps.who.int/iris/handle/10665/69137

[18] Hasso-Agopsowicz H. Global Review of the Age Distribution of Rotavirus Disease in Children Aged <5 Years Before the Introduction of Rotavirus Vaccination. *Clinical Infectious Diseases*, Volume 69, Issue 6, 15 September 2019, Pages 1071–1078, https://doi.org/10.1093/cid/ciz060

[19] Institut National de la Statistique (INS) et ICF International, 2013. Enquête Démographique et de Santé et à Indicateurs Multiples du Niger 2012. Calverton, Maryland, USA : INS et ICF International. Available from https://dhsprogram.com/pubs/pdf/FR277/FR277.pdf Accessed 1 September 2021.

[20] Debellut F. et al. Re-evaluating the potential impact and cost-effectiveness of rotavirus vaccination in 73 Gavi countries: a modelling study. The Lancet Global Health 2019. https://doi.org/10.1016/S2214-109X(19)30439-5

[21] Debellut F. et al. Evaluating the potential economic and health impact of rotavirus vaccination in 63 middle-income countries not eligible for Gavi funding: a modelling study. Lancet Global Health, 2021, https://doi.org/10.1016/S2214-109X(21)00167-4

[22] Clark A. et al. Mortality reduction benefits and intussusception risks of rotavirus vaccination in 135 low-income and middle-income countries: a modelling analysis of current and alternative schedules. Lancet Global Health, 2019, https://doi.org/10.1016/S2214-109X(19)30412-7

[23] Salomon J. A. Disability weights for the Global Burden of Disease 2013 study. Lancet Glob Health. 2015 Nov;3(11):e712-23. https://doi.org/10.1016/s2214-109x(15)00069-8

[24] Clark A, van Zandvoort K, Flasche S, Sanderson C, Bines J, Tate J, Parashar U, Jit M. Efficacy of live oral rotavirus vaccines by duration of follow-up: a meta-regression of randomised controlled trials. The Lancet Infectious Diseases. 2019;19(7):717–727. https://doi.org/10.1016/S1473-3099(19)30126-4

[25] Rogawski ET, Platts-Mills JA, Ross Colgate E, Haque R, Zaman K, Petri WA, et al. Quantifying the Impact of Natural Immunity on Rotavirus Vaccine Efficacy Estimates: A Clinical Trial in Dhaka, Bangladesh (PROVIDE) and a Simulation Study. J Infect Dis Nat Immun Vaccine Effic. JID. 2018:861. https://doi.org/10.1093/infdis/jix668

[26] WHO UNICEF coverage estimates WHO World Health Organization: Immunization, Vaccines And Biologicals. Vaccine preventable diseases Vaccines monitoring system 2020 Global Summary Reference Time Series: DTP3. Available from https://apps.who.int/immunization_monitoring/globalsummary/timeseries/tswucoveragedtp3.html Accessed on accessed 15 September 2021.

[27] Clark A, Sanderson C. Timing of children's vaccinations in 45 low-income and middle-income countries: an analysis of survey data. Lancet. 2009;373(9674):1543–9. https://doi.org/10.1016/s0140-6736(09)60317-2

[28] Gavi, The Vaccine Alliance. Product information for vaccines and cold chain equipment. Detailed product profiles. Available from https://www.gavi.org/news/document-library/detailed-product-profiles Accessed on 9 September 2021

For peer review only

[29] Gavi, the Vaccine Alliance. Co-financing Policy. Version 2.0. Available from https://www.gavi.org/news/document-library/gavi-co-financing-policy Accessed on 9 September 2021.

[30] Gavi, The Vaccine Alliance. Eligibility and Transition Policy. Version 3.0. Available from https://www.gavi.org/news/document-library/gavi-eligibility-and-transition-policy Accessed on 9 September 2021.

[31] WHO. Vaccine Wastage Rates Calculator. 2021. Available from https://www.who.int/publications/m/item/vaccine-wastage-rates-calculator Accessed on 19 October 2021.

[32] World Health Organization. Health Products and Policy Standards. Controlled Temperature Chain (CTC). Beyond the traditional cold chain. Available from https://www.who.int/teams/health-product-and-policy-standards/standards-and-specifications/ctc/ Accessed on 29 October 2021.

[33] Lee B. Update on rotavirus vaccine underperformance in low- to middle-income countries and next-generation vaccines. Human Vaccines Immunotherapeutics 2021; 17(6): 1787–1802. https://dx.doi.org/10.1080%2F21645515.2020.1844525

[34] Palestinian Ministry of Health, Rostropovich Vishnevskaya Foundation, PATH. The health benefits of rotavirus immunization for children in Palestine Results of a vaccine impact analysis. Brief published Sept. 2021. Available from https://path.azureedge.net/media/documents/PATH_Palestine_Rotavirus_Epidemiology_ENGLISH_0d0BYfV.pdf. Accessed on 23 June 2022

[35] Park J., Goldstein J., Haran M., and Ferrari M. An ensemble approach to predicting the impact of vaccination on rotavirus disease in Niger. Vaccine. 2017 Oct 13; 35(43): 5835–5841. https://dx.doi.org/10.1016%2Fj.vaccine.2017.09.020

[36] Debellut F, Jaber S, Bouzya Y, Sabbah J, Barham M, Abu-Awwad F, et al. Introduction of rotavirus vaccination in Palestine: An evaluation of the costs, impact, and cost-effectiveness of ROTARIX and ROTAVAC. PLoS One 2020;15:e0228506. https://doi.org/10.1371/JOURNAL.PONE.0228506.

[37] PATH, The switch from ROTARIX to ROTAVAC in Ghana: Answers to four key questions. Available from https://www.path.org/resources/switch-rotarix-rotavac-ghana-answers-four-key-questions/. Accessed on 23 June 2022.

For peer review only

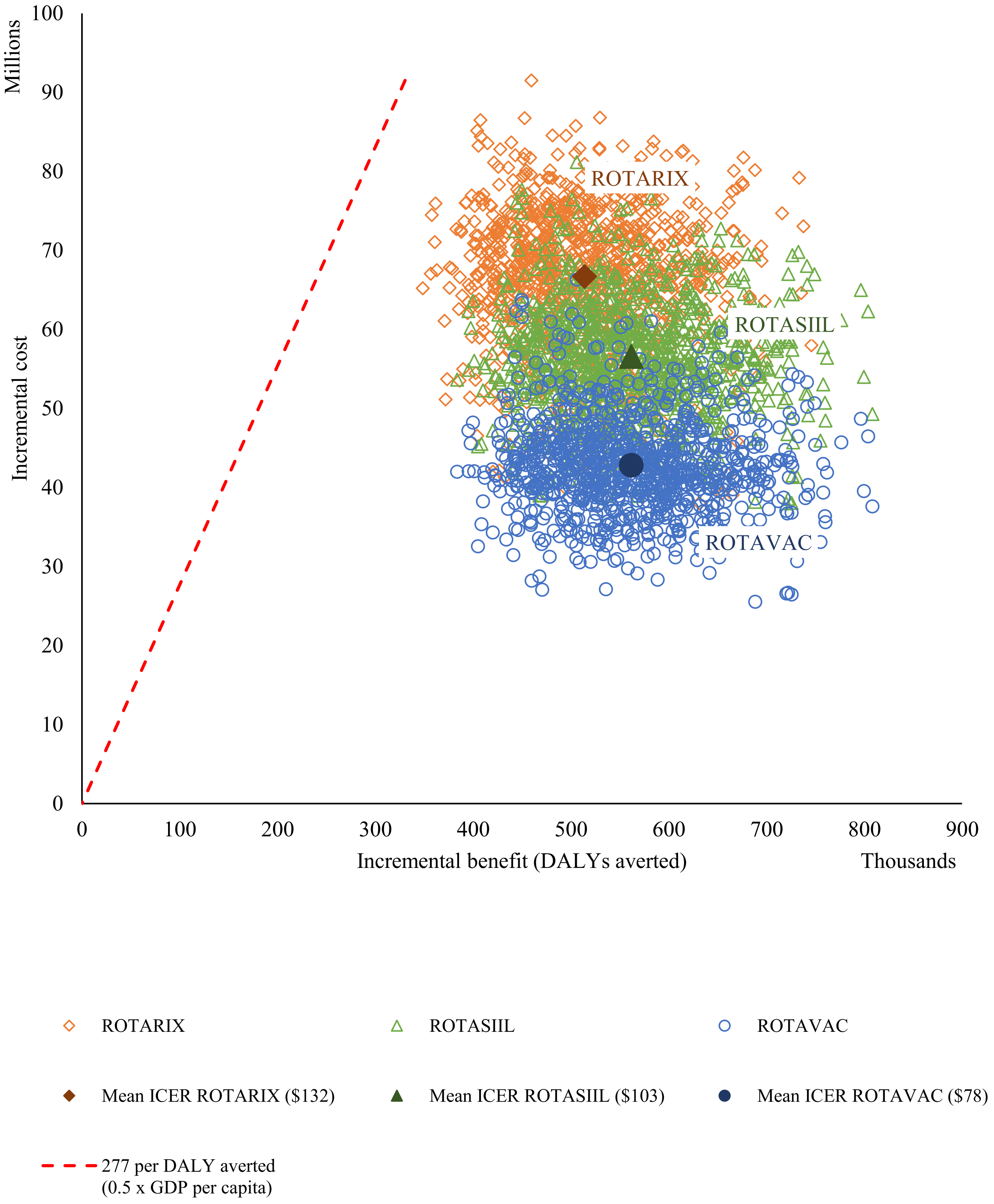
Millions
Incremental cost
100
90
80
70
60
50
40
30
20
10
0
0
100
200
300
400
500
600
700
800
900
Incremental benefit (DALYs averted)
Thousands
ROTARIX
ROTASIIL
ROTAVAC
ROTARIX
ROTASIIL
ROTAVAC
Mean ICER ROTARIX ($132)
Mean ICER ROTASIIL ($103)
Mean ICER ROTAVAC ($78)
277 per DALY averted
(0.5 x GDP per capita)

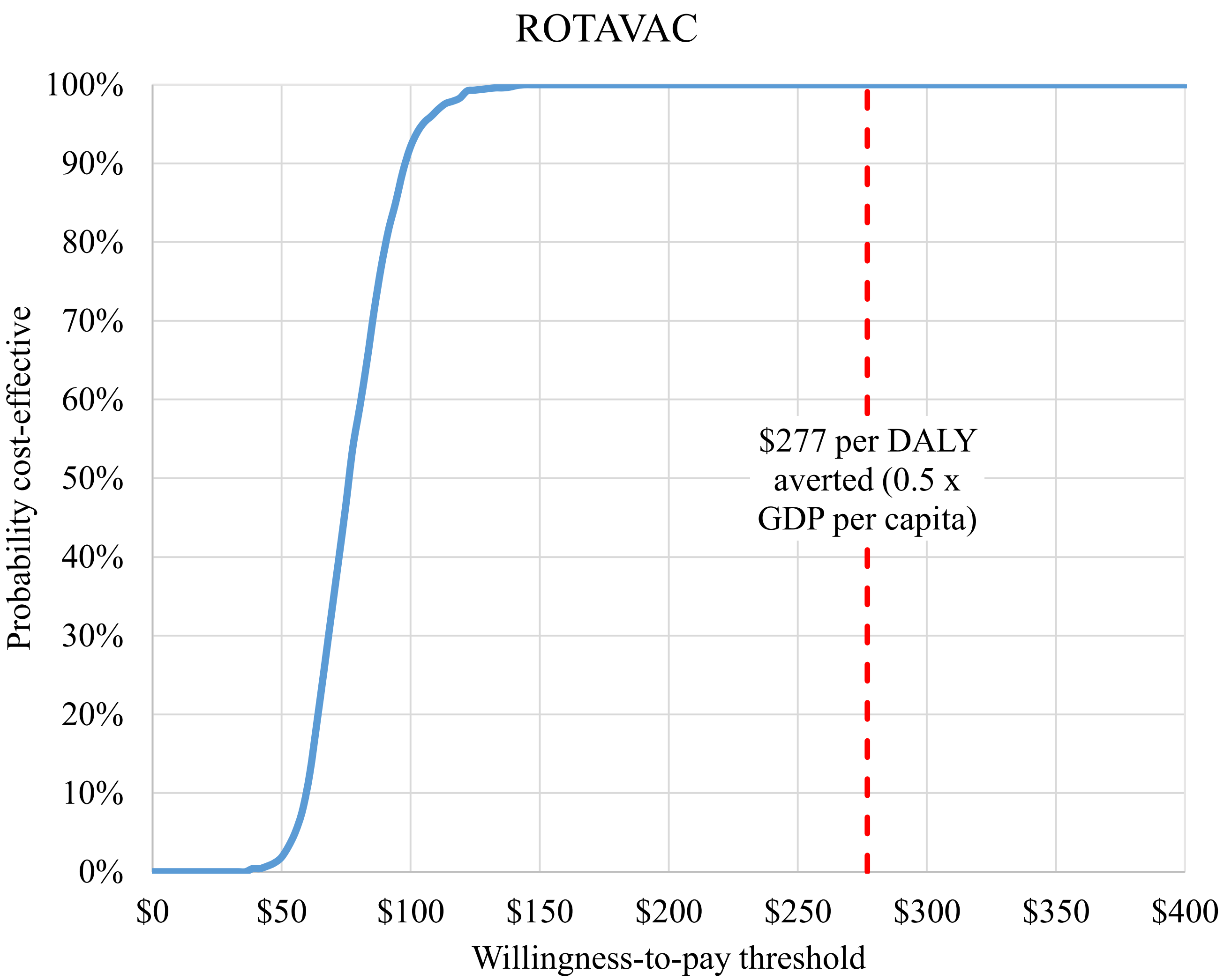
ROTAVAC
100%
90%
80%
70%
60%
50%
40%
30%
20%
10%
0%
Probability cost-effective
$277 per DALY averted (0.5 x GDP per capita)
$0
$50
$100
$150
$200
$250
$300
$350
$400
Willingness-to-pay threshold

Table S1: ROSE trial cost of delivery data

Cost categories	Unit cost	ROTARIX (17.12 cm3)	ROTAVAC (4.2 cm3)	ROTASIIL (10.5 cm3)
International transportation and handling	$0.037 per cm3	$0.633	$0.155	$0.388
Vaccine distribution	$0.018 per cm3	$0.302	$0.074	$0.185
Cold chain storage	$0.010 per cm3	$0.167	$0.041	$0.102
Service delivery	$0.43 per dose	$0.43	$0.43	$0.43
**Total cost per dose**				
	**2018 US$**	**$1.53**	**$0.70**	**$1.11**
	**2021 US$**	**$1.65**	**$0.75**	**$1.20**

For peer review only

Table S2: ROSE trial cost of RVGE data (2021 US$)

Outpatient visits		Inpatient stay	
**Total sample**	**365**	**Total sample**	**26**
% girls	39.2%	% girls	34.6%
% boys	60.8%	% boys	65.4%
Average age	75.72 weeks	Average age	44.65 weeks
Average households' direct medical cost	$0.04	Average households' direct medical cost	$1.05
Average households' direct non-medical costs (food, transportation, day care expenses)	$1.03	Average households' direct non-medical costs (food, transportation, day care)	$3.59
Average indirect cost	$1.31	Average indirect cost	$5.65
**Average household costs**	**$2.37**	**Average household costs**	**$10.30**

For peer review only

Table S3: Parameters included in the probabilistic uncertainty analysis and their statistical distributions

(Base case values and range available from the main paper)

Parameter	Probability distribution	Source
**Population projections for the 2015 birth cohort**		
Population by single age/year between birth and 5·0 years	Beta-PERT (mid = UNPOP medium variant, range = UNPOP low/high variant)	United Nations Population Division. World Population Prospects - 2017 revision. Available from: https://population.un.org/wpp/ Accessed July 14, 2020.
**Disease burden estimates**		Isanaka S. et al. Rotavirus vaccine efficacy up to 2 years of age and against diverse circulating rotavirus strains in Niger: Extended follow-up of a randomized controlled trial. PLOS MEDICINE 2021 https://doi.org/10.1371/journal.pmed.1003655
RVGE incidence rate <5 years<br>Non-severe RVGE visits<br>Severe RVGE visits<br>Severe RVGE hospitalizations	Beta-PERT (mid and range reported in Table 1)	
Severe RVGE mortality rate <5 years	Beta-PERT (mid = Log transformed mean of 3 sources of estimates for Niger, range = 95% CI)	Clark A, Tate J, Parashar U, Jit M, Hasso-Agopsowicz M, Henschke N, et al. Mortality reduction benefits and intussusception risks of rotavirus vaccination in 135 low-income and middle-income countries: a modelling analysis of current and alternative schedules. Lancet Glob Heal. 2019 Nov 1;7(11):e1541–52. https://doi.org/10.1016/S2214-109X(19)30412-7
**Age distribution of RVGE deaths**		
Log Logistic scale parameter	Beta-PERT (mid = best fit for Niger/U5MR stratum, range = 95% CI for Niger/U5MR stratum)	Hasso-Agopsowicz M, Ladva CN, Lopman B, Sanderson C, Cohen AL, Tate JE, et al. Global Review of the Age Distribution of Rotavirus Disease in Children Aged under 5 Years Before the Introduction of Rotavirus Vaccination. Clin Infect Dis. 2019 Jan 28. https://doi.org/10.1093/cid/ciz060
**Disability weights for calcualting DALYs**		
Percentage of healthy time lost whilst living with the disease		
Non Severe RVGE	Beta-PERT (mid = Salomon, GBD 2013, moderate diarhea, range = Salomon, GBD 2013, moderate diarrhea, 95% uncetainty interval)	Salomon JA, Haagsma JA, Davis A, Maertens De Noordhout C, Polinder S, Havelaar AH, et al. Disability weights for the Global Burden of Disease 2013 study. Vol. 3, Articles Lancet Glob Health. 2015. https://doi.org/10.1016/S2214-109X(15)00069-8
Severe RVGE	Beta-PERT (mid = Salomon, GBD 2013, severe diarhea, range = Salomon, GBD 2013, severe diarhea, 95% uncetainty interval)	
Non Severe RVGE	Beta-PERT (mid = ROSE trial, range = ROSE trial)	Isanaka S. et al. Rotavirus vaccine efficacy up to 2 years of age and against diverse circulating rotavirus strains in Niger: Extended follow-up of a randomized controlled trial. PLOS MEDICINE 2021 https://doi.org/10.1371/journal.pmed.1003655
Severe RVGE	Beta-PERT (mid = ROSE trial, range = ROSE trial)	
**Vaccine coverage**		
Doses with DTP1, DTP2, DTP3	Beta-PERT (mid = WUENIC 2020, range = WUENIC 2020 +/-10%)	WHO UNICEF coverage estimates WHO World Health Organization: Immunization, Vaccines And Biologicals. Vaccine preventable diseases Vaccines monitoring system 2020 Global Summary Reference Time Series: DTP3. Available from https://apps.who.int/immunization_monitoring/globalsummary/timeseries/tswucoveragedtp3.html Accessed on accessed 15 September 2021.
**Vaccine timeliness**		
Log Logistic scale parameter	Beta-PERT (mid = best fit for Niger or schedule stratum, range = median age +/-10%)	Clark A, Sanderson C. Timing of children's vaccinations in 45 low-income and middle-income countries: an analysis of survey data. Lancet. 2009;373(9674):1543–9. https://doi.org/10.1016/s0140-6736(09)60317-2
**Vaccine efficacy**		
Initial efficacy against RVGE mortality (2wks after dose administered)	Beta (alpha = 723·19, beta = 98·31, [A] = 0%, [B] = 100%)	Clark A, van Zandvoort K, Flasche S, Sanderson C, Bines J, Tate J, Parashar U, Jit M. Efficacy of live oral rotavirus vaccines by duration of follow-up: a meta-regression of randomised controlled trials. The Lancet Infectious Diseases. 2019;19(7):717–727. https://doi.org/10.1016/S1473-3099(19)30126-4
Mean duration of vaccine efficacy in months	Beta-PERT	
Relative efficacy of 1 dose versus 2/3 doses	Base value: 0·63 (0·51%-0·79%)<br>Beta (alpha = 27·05, beta = 15·68, [A] = 0, [B] = 1)	

Parameter	Probability distribution	Source
**Vaccine program costs and healthcare costs**	Beta-PERT (mid=ROSE costing studies, range = ROSE costing studies)	ROSE costing studies<br>Gavi, the Vaccine Alliance. Co-financing Policy. Version 2.0. Available from https://www.gavi.org/news/document-library/gavi-co-financing-policy Accessed on 9 September 2021.<br>Gavi, The Vaccine Alliance. Eligibility and Transition Policy. Version 3.0. Available from https://www.gavi.org/news/document-library/gavi-eligibility-and-transition-policy Accessed on 9 September 2021.<br>Baral R. et al. Cost of illness for childhood diarrhea in low- and middle-income countries: a systematic review of evidence and modelled estimates. BMC Public Health. 2020; 20: 619. https://dx.doi.org/10.1186%2Fs12889-020-08595-8

For peer review only

Table S4: Health and economic benefits of vaccine (2021-2030, discounted), assuming 2-dose ROTARIX had similar impact to the 3-dose ROTAVAC or ROTASIIL.

**Health and economic benefits**	**ROTARIX (2 doses)**	**ROTAVAC (3 doses)**	**ROTASIIL (3 doses)**
Non-severe RVGE cases averted	774,058	774,058	774,058
Non-severe RVGE visits averted	394,770	394,770	394,770
Severe RVGE cases averted	556,379	556,379	556,379
Severe RVGE visits averted	283,753	283,753	283,753
Severe RVGE hospitalisations averted	140,263	140,263	140,263
Deaths averted	21,631	21,631	21,631
Healthcare cost averted (US$)			
Government perspective	5,062,897	5,062,897	5,062,897
Societal perspective	7,714,859	7,714,859	7,714,859
DALYs averted	544,142	544,142	544,142
Vaccine programme cost (US$)	77,257,820	46,670,448	61,805,765
**Cost-effectiveness ratio (US$ per DALYs averted compared to no vaccine scenario)**			
Government perspective	133	76	104
Societal perspective	128	72	99

For peer review only

Figure S1: Scenario analysis results showing incremental cost-effectiveness ratio (US$ per DALY averted) from the government and societal perspectives of ROTARIX, ROTAVAC, and ROTASIIL compared to no vaccination.

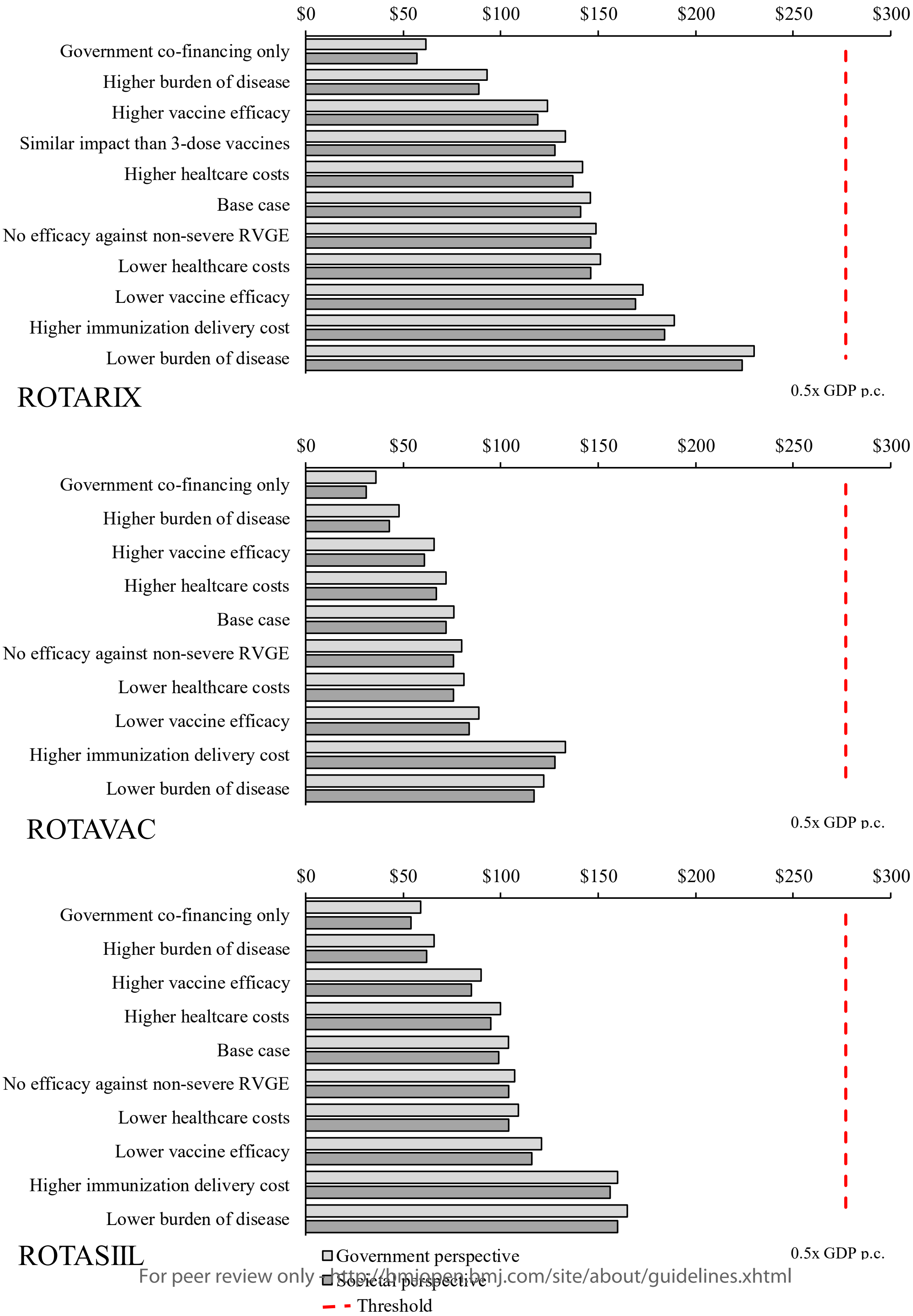

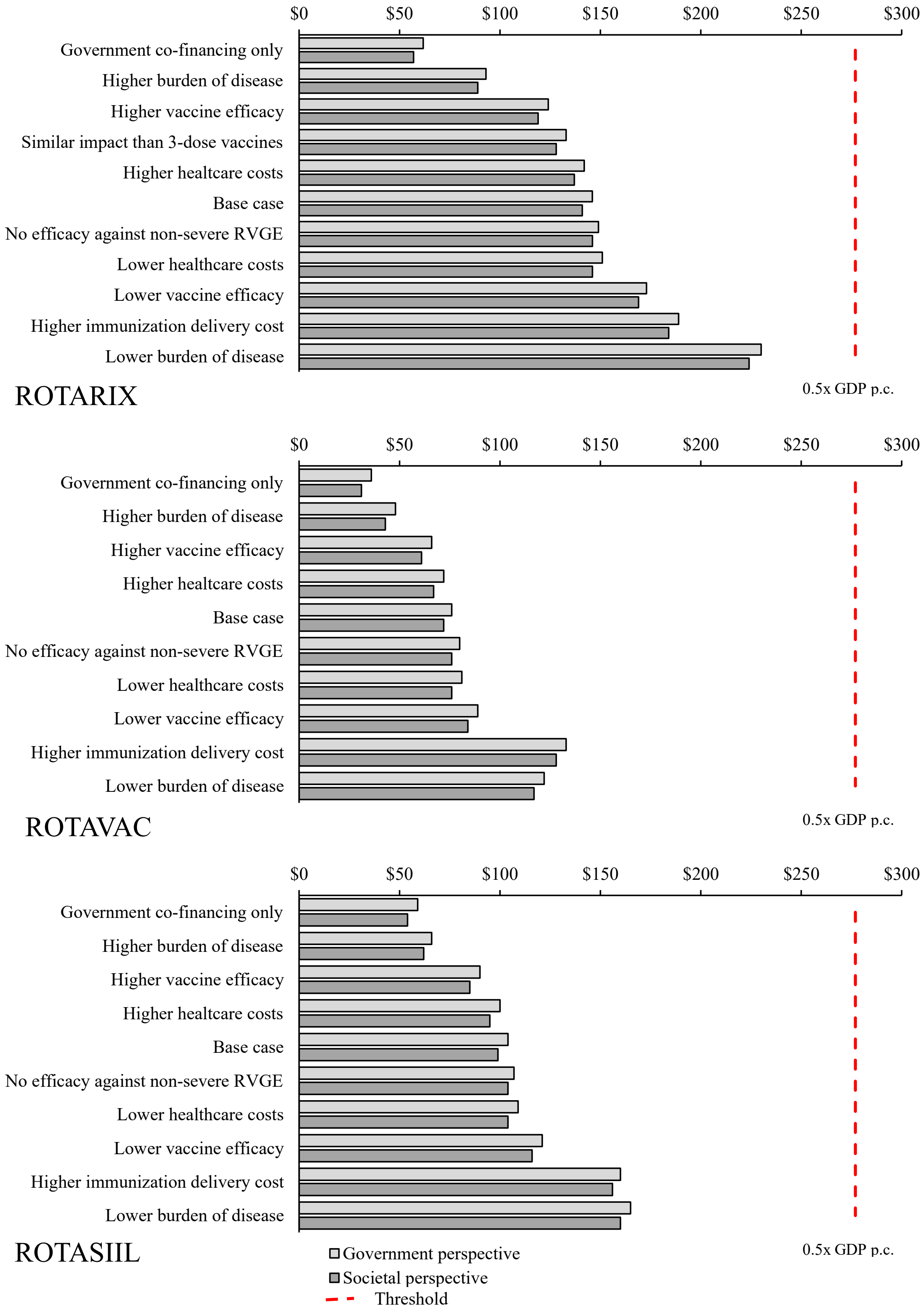
$0
$50
$100
$150
$200
$250
$300
Government co-financing only
Higher burden of disease
Higher vaccine efficacy
Similar impact than 3-dose vaccines
Higher healtcare costs
Base case
No efficacy against non-severe RVGE
Lower healthcare costs
Lower vaccine efficacy
Higher immunization delivery cost
Lower burden of disease
ROTARIX
0.5x GDP p.c.
ROTAVAC
0.5x GDP p.c.
ROTASIIL
Government perspective
Societal perspective
Threshold
0.5x GDP p.c.

ISPOR
Improving healthcare decisions

Consolidated Health Economic Evaluation Reporting Standards (CHEERS) 2022 Checklist

The CHEERS 2022 statement replaces the 2013 CHEERS statement, which should no longer be used. The CHEERS 2022 checklist contains 28 items with accompanying descriptions. Checklist users should indicate the section of the manuscript where relevant information can be found. The authors recommend using a section heading with a paragraph number. If an item does not apply to a particular economic evaluation, checklist users are encouraged to report "Not Applicable." If information is otherwise not reported, checklist users are encouraged to write, "Not Reported." Users should avoid the term "Not Conducted" as CHEERS is intended to guide and capture reporting. Additional information on CHEERS 2022 can be found here.

Title

1. Title

Identify the study as an economic evaluation and specify the interventions being compared.

Abstract

2. Abstract

Provide a structured summary that highlights context, key methods, results, and alternative analyses.

Introduction

3. Introduction: Background and Objectives

Give the context for the study, the study question, and its practical relevance for decision making in policy or practice.

Methods

4. Health economic analysis plan

© 2022 ISPOR—The Professional Society for Health Economics and Outcomes Research
International Society for Pharmacoeconomics and Outcomes Research, Inc

Indicate whether a health economic analysis plan was developed and where available.

---

5. Study population

Describe characteristics of the study population (such as age range, demographics, socioeconomic, or clinical characteristics).

---

6. Setting and location

Provide relevant contextual information that may influence findings.

---

7. Comparators

Describe the interventions or strategies being compared and why chosen.

---

8. Perspective

State the perspective(s) adopted by the study and why chosen.

---

9. Time horizon

State the time horizon for the study and why appropriate.

---

10. Discount rate

Report the discount rate(s) and reason chosen.

---

11. Selection of outcomes

Describe what outcomes were used as the measure(s) of benefit(s) and harm(s).

---

12. Measurement of outcomes

Describe how outcomes used to capture benefit(s) and harm(s) were measured.

© 2022 ISPOR—The Professional Society for Health Economics and Outcomes Research
International Society for Pharmacoeconomics and Outcomes Research, Inc
For peer review only - http://bmjopen.bmj.com/site/about/guidelines.xhtml

ISPOR
Improving healthcare decisions

13. Valuation of outcomes
Describe the population and methods used to measure and value outcomes.

____

14. Measurement and valuation of resources and costs
Describe how costs were valued.

____

15. Currency, price date, and conversion
Report the dates of the estimated resource quantities and unit costs, plus the currency and year of conversion.

---

16. Rationale and description of model
If modeling is used, describe in detail and why used. Report if the model is publicly available and where it can be accessed.

____

17. Analytics and assumptions
Describe any methods for analyzing or statistically transforming data, any extrapolation methods, and approaches for validating any model used.

____

18. Characterizing heterogeneity
Describe any methods used for estimating how the results of the study vary for subgroups.

____

19. Characterizing distributional effects
Describe how impacts are distributed across different individuals or adjustments made to reflect priority populations.

____

20. Characterizing uncertainty
Describe methods to characterize any sources of uncertainty in the analysis.

____ **21.**

21. Approach to engagement with patients and others affected by the study

© 2022 ISPOR—The Professional Society for Health Economics and Outcomes Research
International Society for Pharmacoeconomics and Outcomes Research, Inc
For peer review only - http://bmjopen.bmj.com/site/about/guidelines.xhtml

Describe any approaches to engage patients or service recipients, the general public, communities, or stakeholders (eg, clinicians or payers) in the design of the study.

____

Results

22. Study parameters

Report all analytic inputs (eg, values, ranges, references) including uncertainty or distributional assumptions.

____

23. Summary of main results

Report the mean values for the main categories of costs and outcomes of interest and summarize them in the most appropriate overall measure.

____

24. Effect of uncertainty

Describe how uncertainty about analytic judgments, inputs, or projections affects findings. Report the effect of choice of discount rate and time horizon, if applicable.

____

25. Effect of engagement with patients and others affected by the study

Report on any difference patient/service recipient, general public, community, or stakeholder involvement made to the approach or findings of the study.

____

Discussion

26. Study findings, limitations, generalizability, and current knowledge

Report key findings, limitations, ethical, or equity considerations not captured and how these could impact patients, policy, or practice.

____

© 2022 ISPOR—The Professional Society for Health Economics and Outcomes Research
International Society for Pharmacoeconomics and Outcomes Research, Inc.
For peer review only - http://bmjopen.bmj.com/site/about/guidelines.xhtml

ISPOR Improving healthcare decisions

Health Economic Evaluation ISPOR CHEERS 2022 Reporting Standards

Other Relevant Information

27. Source of funding

Describe how the study was funded and any role of the funder in the identification, design, conduct, and reporting of the analysis.

---

28. Conflicts of interest

Report authors' conflicts of interest according to journal or International Committee of Medical Journal Editors requirements.

© 2022 ISPOR—The Professional Society for Health Economics and Outcomes Research
International Society for Pharmacoeconomics and Outcomes Research, Inc.
For peer review only - http://bmjopen.bmj.com/site/about/guidelines.xhtml
